# Electrostatic tuning of the pyridoxal-5′-phosphate cofactor site defines pH dependence in type I cystathionine β-lyases

**DOI:** 10.1016/j.jbc.2026.111469

**Published:** 2026-04-17

**Authors:** Yuanxiang Liu, Xin Li, Jianxun Li, Jinyan Liu, Yishu Peng, Yan Gao, Ye Zhang, Zijian Jia, Kexin Kang, Anwei Yin, Cuiqing Ma, Yuechao Yang, Chunyu Yang

**Affiliations:** 1 State Key Laboratory of Microbial Technology, Institute of Microbial Technology, Shandong University, Qingdao, P. R. China; 2 College of Resources and Environment, Shandong Agricultural University, Taian, P. R. China; 3 Department of Soil and Water Sciences, Tropical Research and Education Center, IFAS, University of Florida, Homestead, Florida, USA

**Keywords:** thiosulfinate, bacterial alliinase, PLP cofactor, fold-type I PLP-dependent enzymes, residue pair, pH switch

## Abstract

Thiosulfinates, commonly known as “plant antibiotics”, exhibit broad-spectrum antimicrobial activity, but are inherently unstable under alkaline conditions. Thus, the development of biocatalysts that maintain high efficiency at acidic pH is crucial. In this study, we determined the structures of two *patB* gene-encoded cystathionine β-lyases from the fold-type I pyridoxal-5′-phosphate (PLP)-dependent family, BcPatB and LdPatB, which exhibit distinct pH dependencies in catalyzing the conversion of l-cysteine-S-conjugate sulfoxides to thiosulfinates. Structural comparison of these two PatBs revealed a residue pair located near the negatively charged phosphate group of the PLP cofactor, exhibiting distinct charge properties. By mutating this residue pair to enhance negative-negative charge interactions with the PLP phosphate, we generated the BcPatB mutant H263E/Q238M and LdPatB mutant H241M, both of which exhibited significantly improved activity at mildly acidic pH of 6.0. This strategy was subsequently applied to other fold-type I PLP-dependent enzymes, including MePatB, *metC* gene-encoded cystathionine β-lyase from *Klebsiella pneumoniae* (KpMetC), and the alanine aminotransferase from *Escherichia coli* K-12 (Eck-12AlaA), resulting in mutants with modified pH preferences. Constant-pH molecular dynamics simulations demonstrated that the modified electrostatic interactions between PLP and the residue pair play a key role in driving pH-dependent catalysis in these mutants. These findings suggest that this residue pair may function as a pH switch in certain fold-type I PLP-dependent enzymes, and that rational engineering of this position offers a promising strategy for tailoring enzymes to specific industrial pH conditions.

Fold-type I PLP-dependent enzymes are a diverse class of biocatalysts that utilize pyridoxal-5′-phosphate (PLP) as a cofactor. These enzymes can catalyze a wide range of biochemical reactions, including transamination, decarboxylation, racemization, carbon-sulfur (C–S) bond cleavage (β- and γ-elimination) (1, 2, 3), *etc.* The catalytic activity and stability of these enzymes are highly influenced by environmental pH, with optimal pH ranges varying significantly based on the specific function and biological context (4, 5, 6). Understanding the pH dependence of these enzymes is crucial for their effective application in industrial and therapeutic settings, where pH conditions are often variable (7, 8).

Among fold-type I PLP-dependent enzymes, we previously reported BcPatB, a cystathionine β-lyase (CBL) from *Bacillus cereus* (EC 4.4.1.13, encoded by *patB*), as a high-activity alliinase (9). It catalyzes the conversion of l-cysteine-S-conjugate sulfoxides into product thiosulfinates, such as converting l-alliin (S-allyl-l-cysteine sulfoxide) into allicin (diallyl thiosulfinate), a plant antibiotic known for its broad-spectrum antimicrobial properties (Fig. 1*A*). The stability and bioactivity of these labile thiosulfinates are pH-dependent, demonstrating relatively high stability under mildly acidic conditions (pH 5–6) but undergoing rapid hydrolysis at alkaline pH (pH > 7) (10, 11, 12, 13). Although BcPatB exhibits peak alliinase activity (208.9 U/mg for ± l-alliin) at pH 8.0, it retains only 4.6% activity at pH 6.0 (Fig. 1*B*). This optimal pH (pH_opt_) mismatch between the enzyme activity and thiosulfinate stability underscores the significance to identify PatB with robust activity under mildly acidic conditions (Fig. 1) or adjust the acidic-alkaline adaptation of the enzyme toward the acidic direction.

**Figure 1:**
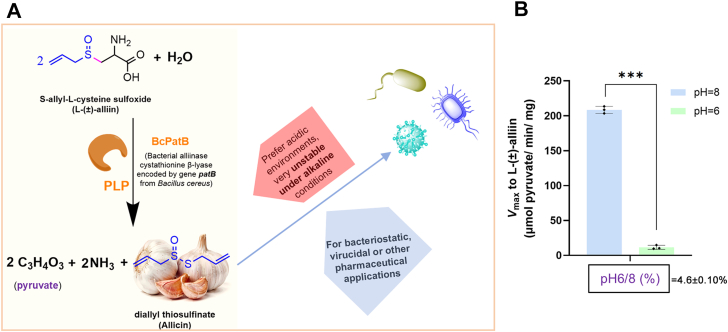
**Schematic diagram of BcPatB used for allicin synthesis and its activity under different pH conditions.***A*, catalysis of l-(±)-alliin to allicin by bacterial alliinase BcPatB. *B*, *V*_max_ of BcPatB measured at pH 6.0 and pH 8.0. The pH 6/8 value (%) represents the ratio of enzyme activity at pH 6.0 to that at pH 8.0 marked below the x-axis, the value > 100% indicates a preference for mildly acidic pH. Statistical significance was determined using an unpaired two-sided *t* test with Welch’s correction. *Asterisks* indicate significance levels: ∗*p* < 0.05 and ∗∗∗*p* < 0.001. Error bars represent the standard deviation from three independent experiments.

Various strategies have been developed to improve the pH adaptation of enzymes in specific environments. Among these, charged residue engineering showed great potential in altering the pH dependence of enzymes. To date, a small fraction of studies on engineering the surface residues (14) or titratable residues within active sites are proved as effective approaches for shifting the pH optimum (15). In this study, based on the resolved structures of two PatBs, BcPatB and LdPatB, which have high alliinase activity (>60 U/mg) but differ in their pH dependence, we identified a structurally conserved residue pair adjacent to the phosphate group of PLP cofactor. Modification of the charged properties of this residue pair remarkably enhanced the mildly acidic activity of these PatBs, likely by modulating charge interactions with the cofactor, a finding further supported by constant-pH molecular dynamics (CpHMD) simulations. In addition, we confirmed the role of this residue pair in altering pH dependence in three other fold-type I PLP-dependent enzymes, with resulting mutants displaying altered pH dependence.

## Results

### Mining PatBs with mildly acidic pH preference

To identify PatBs with alliinase activity preferentially active under mildly acidic conditions, which would benefit product stability, the pH preference of each enzyme was assessed by calculating the activity ratio at pH 6.0 to that at pH 8.0 (pH 6/8); values greater than 100% indicated greater activity at mildly acidic conditions.

**Figure 2:**
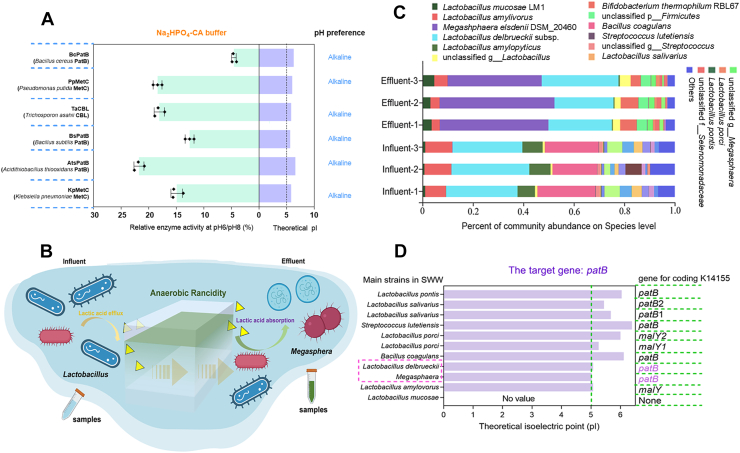
**Identification and characterization of PatBs with acidic pH preference.***A*, theoretical isoelectric points (p*I*s) and mildly acidic pH preference of identified alliinases. Relative enzyme activity at pH 6/8 (%) is calculated to represent mildly acidic pH preference, measured using a Na_2_HPO_4_-Citrate buffer. *B*, schematic representation of the bacteria that formed an acidic intracellular environment in the soy whey wastewater (SWW) microbial community. Specific bacteria may uptake lactate for intracellular metabolism, potentially leading to the formation of an acidic cytoplasm. *C*, species abundance in the SWW influent and effluent samples. *D*, the p*I* values of potential target enzymes with KO number K14155 in high-abundance strains.

Previous studies have established that undissociated organic acids—such as acetic and lactic acids—can diffuse across bacterial membranes when the ambient pH falls below their respective p*K*_a_ values, resulting in localized reductions in cytoplasmic pH (16, 17). Drawing on these insights, we sought to identify acidic alliinases within an organic acid-enriched, anaerobic environment—specifically, soy whey wastewater (SWW)—to target a microbial community that metabolizes organic acids intracellularly. In addition, a review of all CBLs previously investigated by our team revealed that they are all enzymes with alkaline pH preference, coupled with higher theoretical isoelectric points (p*I* > 5.0; Fig. 2*A*). This prompted the adoption of a selection criterion favoring lower p*I* values to identify candidate alliinases with acidic catalytic properties. Metagenomic sequencing of the SWW influent (pH 4.0–4.5) revealed that *Lactobacillus* constituted 54.7% of its microbial community (Fig. 2, *B* and *C*) and actively secreting lactic acid (8.54 g L^−1^) (Fig. S1*A*). In contrast, anaerobic *Megasphaera* flourished in the SWW effluent (pH 3.5–3.8) (Fig. 2*C*) and the lactic acid levels reduced to 4.53 g L^−1^ (Fig. S1*B*), suggesting these *Megasphaera* strains may preferentially uptake lactate and produce other organic acids (18, 19), which likely created a partly mildly acidic intracellular environment conducive to the evolution of acidic enzymes. Based on our previous findings that only CBLs with KO number K14155 (encoded by *patB*) exhibit high alliinase activity toward both l-(±)-alliin and l-(+)-alliin (9), we selected two candidate acidic enzymes with low p*I* values—LdPatB (p*I* 5.07) from *L. delbrueckii* and MePatB (p*I* 5.02) from *Megasphaera* species—for heterologous expression (Fig. 2*D*).

**Figure 3:**
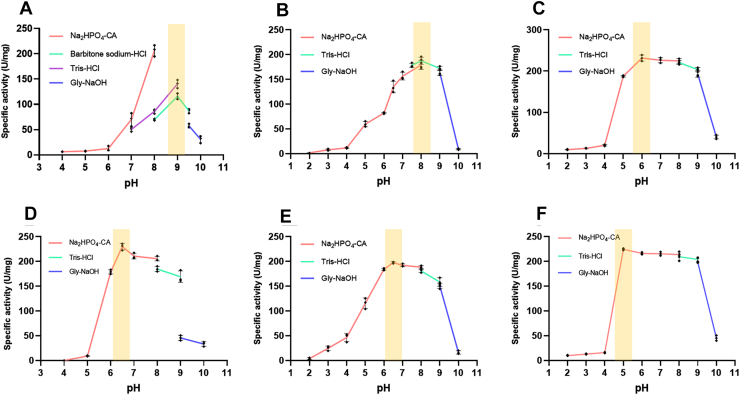
**The optimal pH of three PatBs and their variants.** Specific enzyme activities of BcPatB and BcPatB H263E/Q238M (*A* and *D*), LdPatB and LdPatB H241M (*B* and *E*), MePatB and MePatB F279E (*C* and *F*) under various pH buffers of Na_2_HPO_4_-Citrate (Na_2_HPO_4_-CA), Barbitone sodium-HCl, Tris-HCl, and Gly-NaOH. The *shaded yellow region* indicates the optimal pH range where the inflection point of the specific enzyme activity (U/mg) is observed. Error bars represent the standard deviation from three independent experiments.

Compared to BcPatB, which is nearly inactive at pH 6.0 (Fig. 3*A*), both LdPatB and MePatB exhibited significantly higher activity at pH 6.0 (Fig. 3, *B* and *C*). With a specific activity of 187.4 U/mg, LdPatB has the highest activity at pH 8.0 for l-(±)-alliin, retaining 44.3% of its activity at pH 6.0 (Fig. 3*B*). Notably, MePatB exhibited a pH_opt_ of 6.0 (specific activity 231.4 U/mg) and a pH 6/8 ratio of 103.0% (Fig. 3*C*), indicating superior activity under mildly acidic conditions. Using chemically synthesized l-(±)-alliin as a substrate, which was validated *via* HPLC-MS and hydrogen-1 nuclear magnetic resonance (Fig. S2), the MePatB-catalyzed antimicrobial system exhibited the highest efficiency at pH 6.0. Under 37 °C and initial system pH 6.0, the reaction of 0.23 μg/ml l-(±)-alliin with 0.2 mg/ml purified MePatB completely inhibited the growth of *Escherichia coli* MG1655 (Table S1).

LdPatB and MePatB displayed peak alliinase activity at 60 °C and 65 °C, respectively (Fig. S3, *A* and *B*). Kinetic parameters presented in Table S2 and Fig. S4 confirmed their catalytic properties toward both l-(+)-alliin and l-(±)-alliin substrates at their pH_opt_ conditions. The oligomeric states of BcPatB, LdPatB, and MePatB were determined by sodium dodecyl sulfate–polyacrylamide gel electrophoresis (SDS-PAGE), nonreducing native-PAGE, and size-exclusion chromatography. The results indicated that BcPatB and MePatB were assembled into homotetramers in their protein solutions, whereas LdPatB existed as a homodimer (Fig. S5).

### Three-dimensional structural determination and analysis of PatBs' crystals

**Figure 4:**
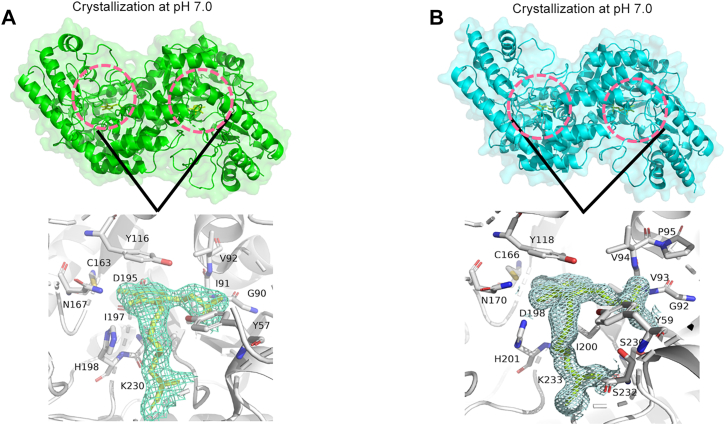
**Cartoon representation and close-up view of BcPatB/PLP and LdPatB/PLP structures.** The PDB IDs for the resolved structures are 9K71 for BcPatB/PLP and 9JVA for LdPatB/PLP. The experimental σA-weighted electron density map (2mFo–DFc) contoured at 1.0 σ. The zoomed in view shows a visible electron density map (*blue mesh*) indicating ligand binding in PatBs. The active site regions within 3 Å of PLP cofactor are highlighted by *pink dashed circles*. *A*, overall structure of BcPatB crystallized at pH 7.0, shown as a *green ribbon diagram* with surface representation. A close-up view of the active sites reveals C4′ of PLP forms an internal aldimine (LLP) with the ε-NH_2_ of K230. *B*, conformation of LdPatB crystallized under identical conditions (pH 7.0), displayed in *cyan*. A close-up view of the active sites reveals C4′ of PLP forms an internal aldimine (LLP) with the ε-NH_2_ of K233. PLP, pyridoxal-5′-phosphate.

In the absence of exogenous PLP, we obtained the LdPatB crystal (Fig. S6) with an open conformation structure, at a resolution of 1.78 Å (PDB ID: 8Y54). After cocrystallized with 0.1 mM PLP, clear electron density for the PLP ligand were detected in both LdPatB and BcPatB structures, with resolutions of 1.56 Å and 2.28 Å, respectively (Fig. 4, *A* and *B*). Crystallographic data collection and refinement statistics are detailed in Tables S3–S5. Despite sharing only 31.2% sequence identity (Fig. S7*A*), the BcPatB/PLP (PDB ID: 9K71) and LdPatB/PLP (PDB ID: 9JVA) complexes demonstrated high structural resemblance, with a low RMSD value of 1.78 Å (Fig. S7*B*). Different from the tetrameric state of BcPatB observed in solution, both crystal forms of PatB assemble as homodimers within each asymmetric unit (Fig. 4, *A* and *B*). As members of the fold-type I PLP-dependent enzyme family, the structures featured a characteristic sandwich structure surrounding the PLP cofactor (20, 21), with the active site residues spanning two chains, requiring to form homodimer to be functional. The sandwich structure comprised conserved residues Y116, PLP, and I197 in BcPatB, which corresponded to Y118, PLP, and I200 in LdPatB (Fig. 4, *A* and *B*). The PLP cofactor was covalently attached to a conserved LYS residue by forming Schiff base—K230 in BcPatB and K233 in LdPatB—with clear electron density for PLP observed at 1.0σ.

The secondary structure analysis of BcPatB revealed that each monomer was composed of 4 β-sheets, 3 βαβ units, 4 β-hairpins, 15 β-strands, 18 α-helices, 19 helix–helix interactions, 25 β-turns, and 3 γ-turns. Similarly, the LdPatB monomer comprised 4 β-sheets, 3 βαβ units, 4 β-hairpins, 1 β-bulge, 15 β-strands, 19 α-helices, 23 helix–helix interactions, 23 β-turns, and 2 γ-turns. Despite structural nuances and low sequence identity (31.2%), both BcPatB and LdPatB adopt a similar overall fold of a Z-shaped conformation, reminiscent of *Allium sativum* alliinase (Fig. S8) (22, 23), which shares 17% and 19% sequence identity with the two enzymes, respectively.

### Structurally conserved residue pairs affecting pH preference in LdPatB and BcPatB

Given that the pH adaptability can be influenced by the charge characteristics of specific residues and the critical role of the PLP cofactor in the catalytic mechanism of PLP-dependent enzymes (24), we performed a comparative structural analysis in the active pockets of LdPatB and BcPatB, focusing on charge interactions between the enzyme and the negatively charged phosphate group of PLP (p*K*_a_ = 2.4) (25).

**Figure 5:**
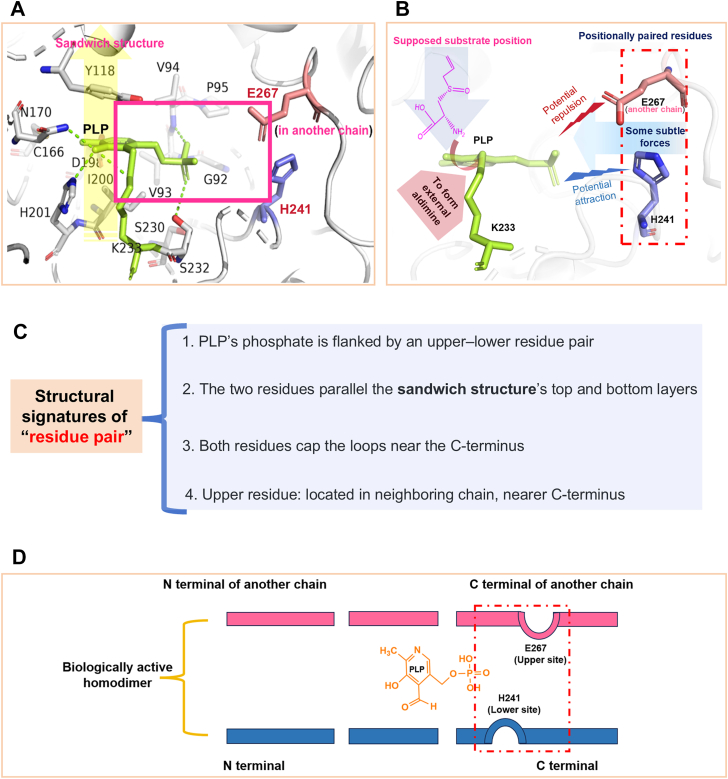
**Structural features of structurally conserved residue pair in LdPatB.***A*, sandwich structure of PLP in LdPatB, with residues involved in this structure highlighted (Y118, PLP, I200). A *red box* encloses two residues, E267 and H241, which are parallel to the *top* Y118 and *bottom* I200 of the sandwich structure, respectively. The PLP molecule is colored in *light green* and the residue pair (H241/E267) is marked in *red*. *B*, proposed structural arrangement at pH 6.0, showing the supposed substrate position and potential interactions between H241 and E267 from adjacent chains. This residue pair may contribute to electrostatic interactions or subtle conformational changes of PLP. *C*, structural features of the residue pair. *D*, simplified two-dimensional view of the residue pair of LdPatB, showing the spatial relationship between paired residues and the phosphate group of PLP. PLP, pyridoxal-5′-phosphate.

In the resolved structures of LdPatB, K233 forms a covalent bond with PLP, creating an internal aldimine, while Y118 and I200 contribute to stabilizing the pyridine ring of PLP (Fig. 5*A*), forming a structure commonly referred to as the “sandwich structure” (26). Notably, we found a pair of residues (E267/H241) located at the C terminus of LdPatB, representing the only charged amino acids flanking the PLP phosphate group. With a p*K*_a_ of 6.47 predicted by PROPKA algorithm, H241 is partially positively charged at pH 6.0, while E267 is negatively charged (with a p*K*_a_ of 4.15) (Fig. 5, *A* and *B*). H241 is positioned at the tip of a loop on the same chain beneath the PLP phosphate group, parallel to the lower layer (I200) of the sandwich structure. E267 is situated at the tip of a loop on the opposite chain, above the PLP phosphate group, parallel to the upper layer (Y118) of the sandwich structure. In contrast, in BcPatB, a partially positively charged H263 occupies a similar position to LdPatB’s E267, while a neutral polar Q238 is spatially aligned with LdPatB’s H241, representing potential paired residues (H263/Q238) in BcPatB (Fig. S9*A*).

Based on the spatial similarity of the loop regions where the residue is situated in LdPatB and BcPatB structures, we infer that these paired residues exhibit structural conservation in their spatial arrangement. Both residues form apex structures of the loop regions and align parallel to the classical sandwich structure (Figs. 5*A* and S9*A*), as summarized in the structural features of LdPatB (Fig. 5, *C* and *D*). However, differences in residue types may lead to significant variations in the electrostatic interactions (attractive or repulsive) formed between these residues and the phosphate group of PLP. We therefore hypothesized that the electrostatic interactions generated between the paired residues and PLP may further influence enzymatic catalytic activity under different pH conditions, as those of H241/E267 shown in Figure 5*B*, which may be one potential explanation for the pH-preference differences observed in BcPatB and LdPatB.

**Figure 6:**
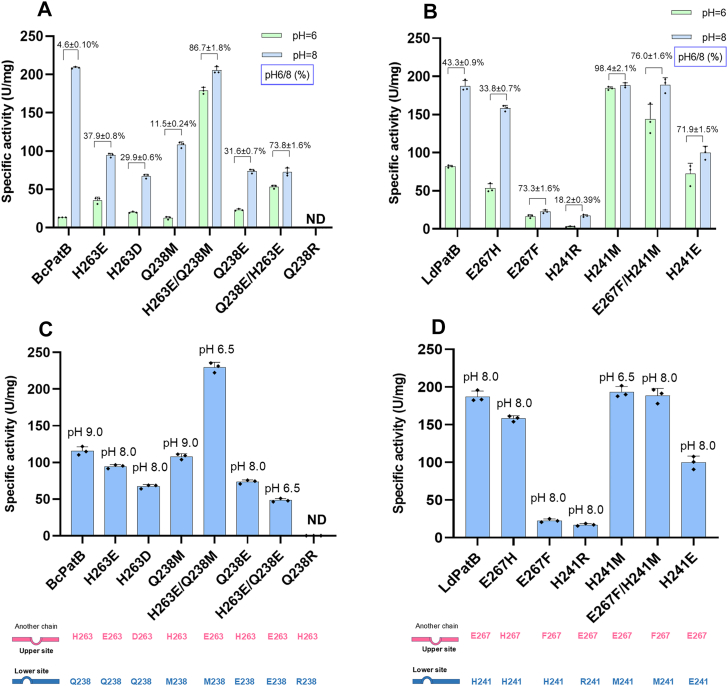
**Mutagenesis of the residue pair in BcPatB and LdPatB.***A* and *B*, mildly acidic pH preference of BcPatB, LdPatB, and their corresponding mutants. The pH 6/8 value (%) represents the specific enzyme activity at pH 6.0 compared to pH 8.0, which is calculated to represent the enzyme’s mildly acidic pH preference. This value is labeled above the column. *C* and *D*, specific activities of BcPatB, LdPatB, and their mutants at their respective pH_opt_. The pH_opt_ values are labeled above each bar. ND indicates that the activity value was not detectable. Error bars represent the standard deviation from three independent experiments.

To investigate the regulatory effects of these structurally conserved residue pairs on pH preferences, we performed site-directed mutagenesis on the corresponding residues of BcPatB (Fig. S9*A*) and LdPatB (Fig. 5*D*). The primary design strategy was to manipulate the electrostatic interactions between the phosphate group of the PLP cofactor and the residue pairs, either by introducing repulsive forces, neutralizing existing attractive forces, or the reverse (Fig. 5*B* and Table S6). The purity of all nickel-nitrilotriacetic acid (Ni-NTA) purified enzymes was >95% as determined by SDS-PAGE and ImageJ software (Fig. S10). The pH preference of the mutant panel revealed several general principles governing the role of these residue pairs. Specifically, replacing positively charged or hydrogen-bond donor residues (*e.g.* H, R/Q) with neutral (M/F) or single negatively charged residues (E/D) shifted the enzyme’s pH_opt_ toward more acidic values, although occasionally at the cost of catalytic efficiency. For example, the BcPatB double mutant H263E/Q238M exhibited a downward shift in pH_opt_ from 9.0 to 6.5 (Figs. 3*D* and 6*C*), along with 14.2-fold increase in specific activity at pH 6.0 (178.9 U/mg) relative to the WT (13.4 U/mg) (Fig. 6*A*). Consistently, this mutant exhibited markedly improved antibacterial activity at pH 6.0. A reaction mixture containing 3.1 μg/ml l-(±)-alliin and 0.2 mg/ml H263E/Q238M completely inhibited *E. coli* growth. In contrast, the wild-type BcPatB required 166.7 μg/ml L-(±)-alliin to achieve similar inhibition (Table S1).

Similarly, the H241M mutation in LdPatB shifted the pH_opt_ from 8.0 to 6.5 (Figs. 3*E* and 6*D*), accompanied by a 2.3-fold increase in specific activity at pH 6.0 (184.2 U/mg for H241M mutant *versus* 81.8 U/mg for the WT) (Fig. 6*B*). In contrast, introduction of additional positively charged residues (R or H) at this position induced an alkaline shift but severely compromised enzymatic activity, such as BcPatB Q238R (Fig. 6, A and *C*) and LdPatB H241R (Fig. 6, *B* and *D*). Worth noticing, excessive negative charge introduction severely diminished the activity, with BcPatB H263E/Q238E only retained 42% activity of the WT level (Fig. 6*C*). Collectively, these observations suggest a principle: modulation of charge interactions with the PLP cofactor can be used to tune pH preference. Introducing repulsive forces or neutralizing existing attractive interactions tends to enhance activity under acidic conditions, whereas reinforcing attractive interactions favors activity under alkaline conditions.

To further probe the uniqueness of the paired residues, we selected BcPatB and mutated other residues within 4 Å of the PLP cofactor to structurally similar acidic or basic amino acids. However, none of these mutations resulted in a shift of the enzyme’s pH_opt_ toward mildly acidic pH or retention of activity (Fig. S11). This outcome supports the conclusion that the identified residue pair plays a unique and critical role in modulating pH preference.

### Validation of the generality of “residue pair” within fold-type I PLP-dependent enzymes

**Figure 7:**
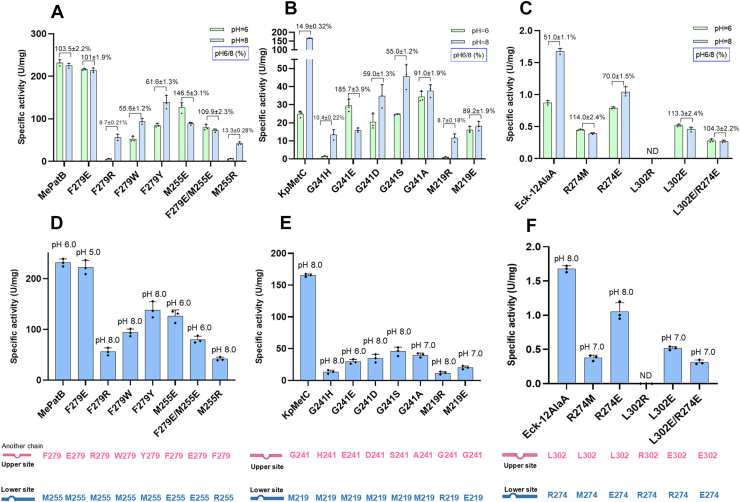
**Mutagenesis of the residue pair in MePatB, KpMetC, and Eck-12AlaA.***A*–*C*, mildly acidic pH preference of MePatB, KpMetC, Eck-12AlaA, and their corresponding mutants. The pH6/8 value (%) represents enzyme activity at pH 6.0 compared to pH 8.0, which is calculated to represent the enzyme’s mildly acidic pH preference. This value is labeled above the column. *D*–*F*, specific activities of MePatB, KpMetC, Eck-12AlaA, and their mutants measured at their respective pH_opt_. The pH_opt_ values are labeled above each bar. ND indicates that the activity value was not detectable. The two residues comprising the residue pair are labeled in *pink* and *blue*, respectively, below the figure. Error bars represent the standard deviation from three independent experiments.

To investigate whether the pH modulation by this residue pair is functional in other fold-type I PLP-dependent members, we selected three additional enzymes of this family for mutation. Since MePatB crystals could not be obtained, an AlphaFold3-predicted MePatB/PLP model with a high pLDDT score (>90) and a stereochemically reasonable conformation (residues within the most favored regions> 90%) was used (Figs. S12 and S13). Structural analysis highlighted the uncharged residues M255 (lower site) and F279 (upper site) as potential structurally conserved residue pair (Fig. S9*B*) and targeted them for mutation. Consistent with the proposed mechanism, altering the charge properties of this residue pair shifted the enzyme’s pH dependence, with MePatB F279E shifting the pH_opt_ from 6.0 to 5.0 (Fig. 3*F*) and the pH5/8 value increasing from 83.6% to 105% (calculated from Fig. 3, *C* and *F*), while maintaining a stable pH6/8 value (101% of F279E *versus* 103% of MePatB) (Figs. 3*F* and 7*A*). Conversely, introducing positive charges (F279R and M255R) reduced both acid-preference and catalytic activity (Fig. 7, *A* and *D*).

PLP binding assays across several key mutants revealed that none showed increased PLP binding relative to WT enzymes (Fig. S14), irrespective of the direction of the pH 6/8 value change. With sufficient PLP supplementation in the reaction mixture, these findings confirmed that the altered pH dependence arose from changes in the enzyme’s response profile, rather than differences in PLP occupancy. Consistently with their improved activities at mildly acidic conditions, all three tested mutants of BcPatB H263 E/Q238M, LdPatB H241M, and MePatB F279E exhibited significantly increased *k*_cat_ values at pH 6.0 or pH 5.0, but not at pH 8.0 (Table 1 and Fig. S15). This indicates that these point mutations indeed prompt the catalytic process of PatBs under mildly acidic conditions. In addition, these enhanced *k*_cat_ values may also support the reversing variations of PLP-occupancy and activity we found in some positive mutations, such as LdPatB H241M, which exhibited reduced PLP occupancy but retained original specific activity. We suppose that such compromise may be contributed by a more active enzyme/PLP complex formed and thus compensated for the decrease in PLP occupancy rate, which is consistent with the increased *k*_cat_ value of H241M detected at pH 6.0 (133.6 s^−1^ *versus* 59.3 s^−1^ of the WT). Collectively, these results support our hypothesis that the residue pair in these PatBs can regulate enzyme activity at different pH, by mainly affecting their catalytic efficiencies. Although we observed variations in their *K*_m_ values (Table 1), no obvious difference was observed between pH 6.0 and 8.0 across every protein pair. We speculate that the shift in PLP position may indirectly influence the geometry of the substrate-binding pocket. However, the lack of substrate-bound structures limits a definitive interpretation of how the residue pair affects substrate affinity and needs to be further addressed.

**Table 1:** Kinetic parameters of PatBs and key mutants toward l-(±)-alliin

pH	Protein	*V*_max_	*k*_cat_ (s^−1^)	*K*_m_ (mM)	*k*_cat_/*K*_m_ (10^3^ M^−1^ s^−1^)
6.0	BcPatB	13.4 ± 0.1	9.7 ± 0.1	26.1 ± 0.6	0.4
	H263 E/Q238 M	178.9 ± 4.3	130.0 ± 3.0	6.6 ± 0.5	19.7 ± 1.5
8.0	BcPatB	208.9 ± 1.2	153.5 ± 0.9	16.7 ± 0.1	9.2 ± 0.1
	H263 E/Q238 M	205.5 ± 4.7	151.0 ± 3.5	2.7 ± 0.3	55.9 ± 6.5
6.0	LdPatB	81.8 ± 1.5	59.3 ± 1.1	3.3 ± 0.2	17.9 ± 1.1
	H241 M	184.2 ± 2.1	133.6 ± 1.5	3.9 ± 0.1	34.3 ± 0.9
8.0	LdPatB	187.4 ± 7.6	137.5 ± 4.9	11.4 ± 1.3	12.1 ± 1.5
	H241 M	188.5 ± 3.0	141.4 ± 2.3	5.2 ± 0.4	27.2 ± 2.1
5.0	MePatB	187.7 ± 2.0	145.5 ± 1.6	7.8 ± 0.2	17.4 ± 0.5
	F279 E	224.5 ± 1.8	174.0 ± 1.4	3.5 ± 0.5	46.5 ± 6.7
8.0	MePatB	222.7 ± 3.4	172.6 ± 2.6	14.4 ± 0.6	12.0 ± 0.4
	F279 E	213.9 ± 2.9	165.8 ± 2.3	15.1 ± 0.8	11.0 ± 0.7

The reaction mixture contains 0.1 mM PLP, 0.02 to 0.1 mg/ml purified protein, substrate l-(±)-alliin, and reacted at 35 °C and specified pH conditions. The buffer used for activity measurement is Na_2_HPO_4_-Citrate (Na_2_HPO_4_-CA). The theoretical molecular weights (Mw) of monomeric BcPatB, LdPatB, and MePatB are 44.1, 45.0 and 46.5 kDa, respectively, calculated using the ProtParam tool on the ExPASy server. The unit of *V*_max_ is μmol pyruvate min^−1^ mg^−1^ protein.

Furthermore, two other fold-type I enzymes with low homology with PatB (<20% identity), KpMetC (CBL encoded by the *metC* gene in *Klebsiella pneumoniae*) we identified previously (9), and Eck-12AlaA (alanine aminotransferase from *E*. *coli* K-12), were tested for the residue pair function. The three-dimensional structure of KpMetC/PLP was modeled using AlphaFold3 and subjected to pLDDT and Ramachandran plot analysis (Figs. S16 and S17), while the Eck-12AlaA/PLP structure was sourced from the Protein Data Bank (PDB; ID: 4CVQ) (27). Guided by the established structural rules, G241/M219 in KpMetC and L302/R274 in Eck-12AlaA were identified as putative structurally conserved residue pair (Fig. S9, *C* and *D*) and targeted for mutation. Consistent with findings on shift of pH_opt_ in PatBs, the G241E mutation in KpMetC shifted the pH_opt_ from 8.0 to 6.0 and increased the pH 6/8 value from 14% to 184%, along with 1.2-fold specific activity at pH 6.0 (30.0 U/mg for G241E *versus* 25.2 U/mg for KpMetC) (Fig. 7, *B* and *E*). Similarly, in Eck-12AlaA, the R274M mutation caused a pH_opt_ reduction from 8.0 to 7.0, with a doubling of the pH 6/8 value (114%) compared to WT (51%), and the L302E mutation also increased the pH 6/8 value (113.3%) (Fig. 7*C*). However, we observed that these three mutations all led to a reduction in specific activity (Fig. 7, *E* and *F*). Conservation analyses indicated that, unlike the variable properties of the residue pairs in three PatBs (Fig. S18, *A–C*), the corresponding residues in KpMetC and Eck-AlaA were highly conserved within their respective enzyme families (Fig. S18, *D* and *E*). This conservation may explain the greater loss of activity observed in these mutants. Nevertheless, the overarching rule remains that removal of a positive charge or addition of a negative charge at this site promotes acidic preference, while introducing an additional positive charge abolishes activity. This principle appears generally applicable in some fold-type I PLP-dependent enzymes.

### CpHMD simulations of LdPatB H241M, BcPatB H263E/Q238M, and Eck-12AlaA R274E

**Figure 8:**
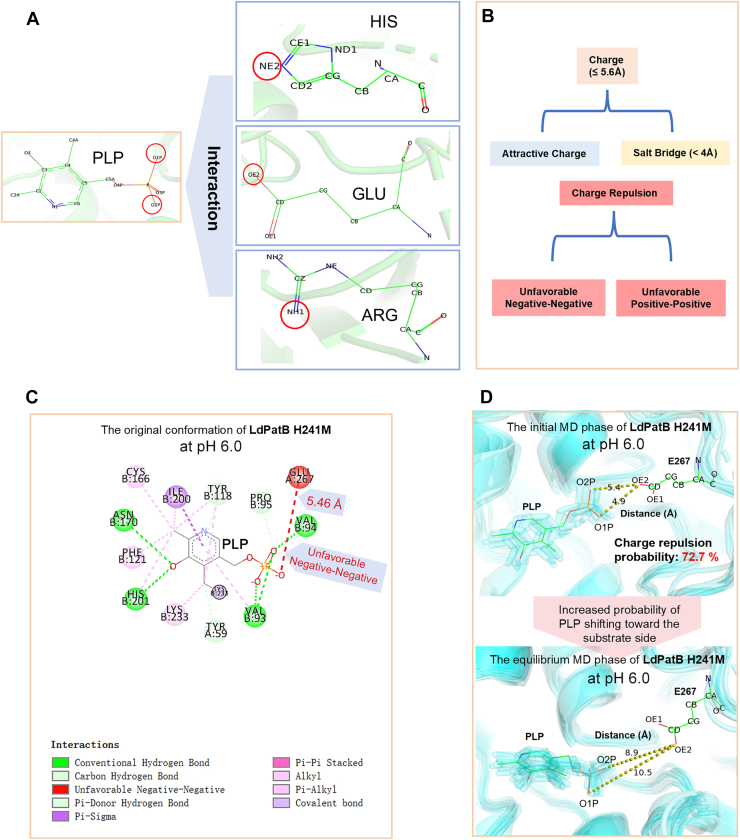
**Constant**-**pH molecular dynamics simulations of LdPatB H241M at pH 6.0.** Clustered conformations of PLP and the switch residues are drawn as sticks; the average interatomic distances after clustering are marked by *yellow dashed lines*. *A*, atoms of PLP and target residues subjected to analysis. The relevant atoms are highlighted with *red circles*. At pH 6.0, the oxygen atoms O1P and O2P in the phosphate group of PLP deprotonate and acquire negative charges. If the atomic distances are appropriate, the OE2 group of GLU will repel O1P and O2P of PLP through charge repulsion, while the NE2 of HIS and NH1 of ARG will attract these atoms through charge attraction. *B*, types of interactions examined. The force-evaluation criteria were adopted from Discovery Studio software. *C*, original conformation (frame 0) of mutant LdPatB H241M. The charge repulsion (Negative-Negative) between E267 and PLP is highlighted in *red* along with the bonding distance (*yellow dash line*). *D*, initial MD phase (frames 0–10) and equilibrium MD phase (frames 175–200) of mutant LdPatB H241M. *Upper* panel shows a close distance (5.4 Å) between O1P of PLP and OE1 of E267, and a 72.7% probability of forming charge repulsion in the initial phase; *lower* panel shows the distance increased to 8.9 Å in the equilibrium phase. PLP, pyridoxal-5′-phosphate.

To gain deeper insights into the molecular mechanisms by which specific residues influence pH preference in PLP-dependent enzymes, we constructed homodimer models (minimal catalytic unit) for several mutants‒BcPatB H263E/Q238M, LdPatB H241M, and Eck-12AlaA R274E‒alongside their respective WT enzymes. Subsequently, CpHMD simulations, which can dynamically modulate charge distributions and predict the electrostatic interactions in solution accurately (28), were performed at pH 6.0 for these models. After the total energy and RMSD values reached a plateau (Fig. S19), principal component analysis was performed on conformations from the initial state (simulation frames 0–10) and the equilateral state (frames 175–200). We then compared the charge interactions between the PLP phosphate group and the residue pair (Fig. 8, *A* and *B*) within the dominant conformations of these mutants and their WTs. CpHMD analysis of mutant LdPatB H241M and LdPatB revealed that the His-Met mutation at pH 6.0, which eliminated the potential electrostatic attraction, obviously repelled the PLP phosphate group from the residue pair E267/M241 (Figs. 8, *C* and *D* and S20). Specifically, in the mutant LdPatB/H241M, the distance between E267 OE2 and PLP O2P began at 5.4 Å, and negative–negative charge clashes were present in 72.7% of early MD simulation frames (Fig. 8*D*). As the simulation progressed into the equilibrium phase, this negatively charged repulsion pushed PLP far beyond 5.6 Å (>8.9 Å) from E267 OE2, and likely drove PLP swing to the substrate side (Fig. 8*D*). This confirmed our hypothesis that the replacement of a positive charged residue with uncharged residue can eliminate the electrostatic attraction to PLP phosphate and subsequently enlarge the distance between the residue pair and PLP.

Similarly, CpHMD analysis of mutant BcPatB H263E/Q238M and BcPatB revealed that the His–Glu mutation at pH 6.0, which eliminated the potential electrostatic attraction and added new charge repulsion, repelled the PLP phosphate group from the residue pair E263/M238. Compared to BcPatB (Fig. S21, *A* and *B*), the negatively charged repulsion between E263 OE2 and PLP O1P/O2P in mutant BcPatB H263E/Q238M persisted throughout both the initial and equilibration phases of the MD simulation. In the dominant conformation during the equilibration phase, the distance between the E263/M238 residue pair and PLP also increased (distance between E263 OE2 and PLP O1P increased from 3.1 Å to 5.0 Å) (Fig. S21, *C* and *D*). Consistently, the Arg‒Glu mutation (R274E) in Eck-12AlaA obviously eliminated the electrostatic attraction and introduced new charge repulsion. Compared to the 100% frames of a charge attraction between R274 NH1 and PLP O1P/O2P of Eck-12AlaA (Fig. S22, *A* and *B*), mutant R274E converted this charge attraction to charge repulsion in 90.9% of the frames in initial state of CpHMD simulation, and consequently we observed an enlarged distance during the equilibration phase, increasing from 5.0 Å to 10.4 Å (Fig. S22, *C* and *D*).

## Discussion

Based on structural comparisons of BcPatB and LdPatB, two PatBs with different pH adaptivity, we identified a charged residue pair near the phosphate group of PLP. Site-directed mutagenesis on these residue pairs resulted in a significant improvement in their alliinase activity under acidic pH conditions. Specifically, the mutants BcPatB H263E/Q238M and LdPatB H241M successfully shifted their pH_opt_ from alkaline values of pH 9.0 and 8.0, respectively, to mildly acidic pH 6.5, while maintaining high catalytic efficiency. This effectively addressed the production challenge arising from the mismatch between the pH_opt_ for thiosulfinate formation and catalytic reaction. Notably, the functional efficacy of this residue pair was also validated in other fold-type I PLP-dependent enzymes, KpMetC and Eck-12AlaA. Thus, we hypothesize that this residue pair may serve as a pH switch for engineering the pH-dependence in certain fold-type I PLP-dependent enzymes. Considering the spatial arrangement and physicochemical properties of the region, this pH switch may fulfill several distinctive structural features: (i) within the homodimeric unit of type-I PLP-dependent enzymes, the switch comprises a pair of residues positioned above and below the negatively charged phosphate group of PLP; (ii) these two residues are aligned parallel to the upper and lower layers of the PLP sandwich structure; and (iii) at the primary structure level, the upper switch residue is located closer to the C terminus of its polypeptide chain than the lower residue (Fig. 5, *C* and *D*).

We initially hypothesized that this residue pair modulates pH-dependent activity by influencing its electrostatic interaction with the PLP phosphate group. The validity of this hypothesis is supported by several panel mutations introducing residues with varying charges, as well as CpHMD simulations of positive mutants exhibiting enhanced acidic pH-preference. Overall, the mutations resulted in a clear increase in the distance between the residue pair and the PLP phosphate group, as the simulation progressed to equilibrium at pH 6.0. This suggests the occurrence of repulsive interactions due to negative charges, which may push PLP away from the residue pair. This altered repulsion aligns well with our initial design principles, where removing a positive charge or adding a negative charge at this site promotes the enzyme’s acidic preference. For example, the native residue pair E267/H241 in LdPatB carries an overall weak negative charge. At pH 6.0, the imidazole group of H241 is partially protonated, while the γ-carboxyl group of E267 is fully deprotonated, generating repulsive charge-charge interactions with PLP. When H241 is replaced by the neutral M241, the negative charge intensity of the E267/M241 pair is increased, resulting in enhanced electrostatic repulsion toward the PLP molecule, as observed in the CpHMD simulations (Fig. 8*C, D*, S20). Notably, PLP exhibits a clear shift toward the substrate-binding cavity in the H241M mutant (Fig. 8*D*). This movement of PLP during external aldimine formation in PLP-dependent enzymes has been documented in previous protein crystallography studies (29). Therefore, we suppose that the conformational shift and movement of PLP toward the substrate cavity may strengthen the interaction between the substrate and cofactor, thereby facilitating the formation of the external aldimine (PLP-substrate) complex. In addition, mutations can induce conformational changes that reduce *K*_m_ values in some mutants, potentially boosting catalytic efficiency by increasing substrate availability within the cavity. These alterations collectively contribute to the higher *k*_cat_ values and improved specific activities observed in the mutants under mildly acidic conditions.

In addition, the shift in the enzyme’s pH preference may also be attributed to alterations in the p*K*_a_ values of a crucial aspartate residue in PLP-dependent enzymes, that is, D198 in LdPatB, D195 in BcPatB, and D233 in Eck-12AlaA. This Asp is strictly conserved across these PLP enzymes and is responsible for stabilizing the protonated pyridinium nitrogen (N1^+^) of PLP *via* a strong hydrogen bond of Asp (β-COO^-^) H–N1^+^ (PLP) (30). This interaction stabilizes the positive charge on the pyridine ring of PLP, and consequently significantly depletes the electron density at the C4′ of PLP, through a conjugated electron-withdrawing effect transmitted *via* the π-electron system. The C4′ also serves as the electrophilic center in the internal aldimine (PLP-Lys; C4=N-Lys), rendering it more electron-deficient (*i.e.*, enhancing its electrophilicity). This increased electrophilicity facilitates the nucleophilic attack by the deprotonated α-amino group (α-NH_2_) of the substrate, also leading to the formation of the external aldimine (31, 32). Therefore, the highly conserved Asp may act as a “nucleophile” in these PLP-dependent enzymes and crucial for the external aldimine formation. Considerable findings revealed that alteration of charged residues in the active center can affect the protonation state of active residues, thus changing their p*K*_a_ values and further influence the pH optimum of enzymes (15, 33, 34). For instance, mutation of Q13E near the active site of pullulanase decreased the p*K*_a_ value of the nucleophile E290 and led to a lower pH_opt_ value of the enzyme (15). Comparing the p*K*_a_ values of this Asp revealed a high consistency with the acid pH alternation of generated mutants, in which the p*K*_a_ of D195 in BcPatB decreased from 6.33 to 5.92 after the double mutation of H263E/H241M, D198 in LdPatB reduced from 6.06 to 5.83 in mutant LdPatB H241M, and D233 changed from 5.79 to 5.54 in mutant Eck-12AlaA R248M. This indicates that this Asp residue in these mutants is more prone to deprotonation at pH 6.0. Consequently, the essential β-COO^-^ form required to stabilize the pyridinium N1^+^ of PLP is more readily established at this pH, ultimately enhancing the electrophilicity of the C4′ carbon in the internal aldimine (C4′ = N-Lys) and facilitating the formation of the external aldimine. In summary, the decreased p*K*_a_ allows the conserved Asp to adopt the necessary negatively charged form under more acidic conditions, thereby may serve as another factor for broadening the acidic catalytic range of these PLP-dependent enzymes.

## Experimental procedures

### Regents, media, and buffers

The substrate l-(±)-alliin was synthesized in our laboratory using established protocols (9). In addition, l-(+)-alliin was obtained from YuanYe Biotech Co., Ltd. Detailed compositions and formulations of the culture media and protein buffers used in this study are provided in Table S7.

### Antibacterial assays

The antimicrobial assay against *E. coli* MG1655 was determined using the double dilution method in a 96-well plate at 37 °C, following the CLSI standard (35). Each well contained 100 μl of MH broth (pH 6.0 or 8.0) system, 20 μg of purified bacterial alliinase, diluted l-(±)-alliin substrate, and 5 × 10^5^ CFU/well of bacterial cells. Two negative controls were included: one lacking l-(±)-alliin and the other without alliinase.

### Metagenomic sequencing

To prepare samples for sequencing, precipitates from SWW influent and effluent were collected by centrifugation at 10,000*g* for 10 min, rapidly frozen in liquid nitrogen, and then subjected to metagenomic sequencing. Amplification was performed on an ABI GeneAmp 9700, and sequencing was performed on an Illumina MiSeq PE 300 high-throughput sequencer (36) (Majorbio Corp). All high-throughput sequencing data were analyzed using the Majorbio I-Sanger cloud platform (http://www.i-sanger.com/). The data have been deposited in NCBI under accession number BioProject PRJNA1012830.

### Protein expression and purification

All primers used were listed in Table S8. Target protein genes were heterologously expressed in *E. coli* BL21(DE3) using the pET-24a(+) expression vector. Detailed information regarding the names and sequences of expressed genes is provided in the Supporting information. Strains were cultured in LB broth, and protein expression was induced with 0.5 mM IPTG, followed by incubation at 20 °C for 10 h. After centrifugation at 6000*g* for 10 min, cells were resuspended in a lysis buffer (Table S7), disrupted, and the lysates were collected by centrifugation to obtain the crude enzyme solution. Crude enzymes were purified using immobilized metal affinity chromatography on a Ni-NTA column, performed on an automated protein purification system (Shanghai Flash Spectro protein purification system). Crude enzyme solution filtered through a 0.22 μm membrane filter was loaded onto the equilibrated Ni-NTA column, followed by extensive washing with binding buffer containing 20 to 40 mM imidazole to remove nonspecifically bound contaminants. Elution was carried out with a linear gradient of imidazole (up to 500 mM), and fractions corresponding to the peak apex of the A_280nm_ absorbance trace were collected to ensure high purity and homogeneity. The eluted protein was subsequently buffer-exchanged into the low-salt buffer using Amicon Ultra centrifugal filter units with a 30 kDa molecular weight cutoff. Final protein purity was assessed by SDS-PAGE followed by Coomassie Brilliant Blue staining and ImageJ software, which confirmed >95% purity with single dominant band matching the expected molecular weight. Protein concentration was determined by Bradford method using bovine serum albumin as standard (37).

### Site-directed mutagenesis

Site-directed mutagenesis was performed using the QuickChange method (38). The recombinant plasmid containing the WT enzyme was extracted from *E. coli* DH5α and used as the template for PCR amplification with primers listed in Table S8. After digestion with DpnI endonuclease at 37 °C for 30 min to remove the methylated DNA template, the PCR product was transformed into *E. coli* DH5α. The mutated plasmids were then isolated, verified by sequencing, and further transformed into *E. coli* BL21(DE3).

### Enzyme activity assays

Alliinase activities of PatBs and KpMetC were measured as previously described (9), using either commercial l-(+)-alliin or synthesized l-(±)-alliin as substrates. Both substrate types were used to determine the kinetic parameters of MePatB and LdPatB, while all other enzymatic assays used l-(±)-alliin exclusively. For specific enzyme activity assays of various mutants, 180 mM l-(±)-alliin, 0.1 mM PLP, and 0.01 to 0.1 mg Ni-NTA purified enzyme were added to a 1-mL reaction system at 35 °C. The specific activities of Eck-12AlaA and its mutants were also assessed using the DNPH colorimetric method with minor modifications (39), in which 0.1 mM PLP, 200 mM L-alanine, 2 mM α-ketoglutarate, and 0.1 mg Ni-NTA purified enzyme were added to a 1-mL reaction system, and reacted at 37 °C for 10 min. One unit of enzyme activity was defined as the amount of enzyme required to generate 1 μmol of pyruvate per minute (1 U = 1 μmol pyruvate min^−1^). One unit of enzyme activity was defined as the amount of enzyme required to generate 1 μmol of pyruvate per minute (1 U = 1 μmol pyruvate min^−1^).

### Crystallization and structure determination

Crystallization of LdPatB and BcPatB purified by Ni-NTA affinity and size-exclusion chromatography (9 mg/ml) was performed using the hanging drop vapor diffusion method. Before crystallization, 1 mM of PLP was added to the protein solution. Equal volumes (1 μl) of protein and reservoir solutions were mixed in sitting-drop plates, with screening conducted across 960 commercial kit conditions. The crystallization process was carried out at 18 °C. Crystals of LdPatB and BcPatB formed in reservoir solutions containing 0.2 M magnesium formate dihydrate and 15 to 25% (w/v) polyethylene glycol 3350 at pH 7.0.

For X-ray diffraction, 20% (v/v) glycerol was used as a cryoprotectant. Diffraction data were collected using an ADSC QUANTUM 315r detector at beamline BL02U1 of the Shanghai Synchrotron Radiation Facility. Data processing, including indexing, integration, and scaling, was performed using the HKL3000 software. Initial structural models of LdPatB and BcPatB were generated *via* molecular replacement using Phaser, followed by further refinement with WinCOOT and Phenix software. All structural images were generated with PyMOL (www.pymol.org/) and Discovery Studio (www.3ds.com/products/biovia/discovery-studio).

### CpHMD simulations

CpHMD simulations were performed on the WeMol Cloud platform (wemol.wecomput.com) using the GROMACS (Version 2024.6) module (40), amber03 force field, and tip3p water model. Given that the active site of type I fold PLP-dependent enzymes spans two subunits, with the minimal functional unit being a homodimer, we chose the homodimeric form to perform simulations. Homodimer mutant models (BcPatB Q238 M/H263 E/PLP, LdPatB H241 M/PLP, and Eck-12AlaA R274 E/PLP) were constructed using WinCOOT based on their respective WT crystal structures of BcPatB (9K71), LdPatB (9JVA), and Eck-12AlaA (4CVQ). Before simulations, all water molecules and ions were removed. The initial conformations (frame 0) were derived from protein protonation, GMX receptor and ligand parameterization, and MD solutions. Protonation was carried out at pH 6.0. Simulations were run for 200 ns with 2 fs time step. Each system was placed inside a cubic box measuring 10 Å per side, with periodic boundary conditions applied and 0.15 M NaCl included. The simulations were conducted under NPT conditions at 300 K and 1.01325 bar. Snapshots of proteins and ligands were taken every 1 ns, yielding 200 frames for trajectory analysis. Clustering analysis using the Gromos algorithm was performed on initial (frames 0–10) and equilibrium (frames 175–200) conformations. For each cluster, the average distances between PLP oxygen atoms (O1P, O2P) and key residues (GLU-OE2, HIS-NE2, ARG-NH1) were calculated. Charge interactions were assessed based on the criteria shown in Figure 8*B*, and the probabilities of charge repulsion and attraction were determined from the interatomic distances in MD frames. Charge interactions in the initial conformations of the three mutants with PLP were assessed and visualized using Discovery Studio (https://www.3ds.com/products/biovia/discovery-studio). Structures from the remaining MD simulations were visualized in PyMOL.

### Analysis and drawing

The drawing tools used include PowerPoint, GraphPad Prism, and Pymol. Protein complex models of KpMetC and MePatB with PLP were generated using AlphaFold3 (its open-source algorithm is executed by the WFold module in the WeMol Cloud Platform. wemol.wecomput.com), with validation *via* Ramachandran plots on the SAVES server (https://saves.mbi.ucla.edu/) and PDBsum database (https://www.ebi.ac.uk/thornton-srv/databases/pdbsum/). Secondary structure analysis was performed by PDBsum. The theoretical p*I* values of enzymes were calculated using the Expasy platform (https://web.expasy.org/compute pi/). The theoretical molecular weight of the monomeric protein was calculated using the ProtParam tool on the ExPASy server (https://web.expasy.org/protparam/). Evolutionary conservation was assessed using ConSurf (41) using the default HomoloGene pipeline (150 sequences, E-value ≤ 10^-3^, sequence identity 35–95%). The p*K*_a_ values of selected ionizable amino acid of enzymes were calculated using PROPKA3 version 3.4.0 (42).

## Data availability

All data supporting the findings of this study are available from the corresponding author upon request.

## Supporting information

This article contains supporting information (22, 27, 41, 43, 44, 45, 46, 47).

## Conflict of interest

The authors declare that they have no conflicts of interest with the contents of this article.
