# Supplementary material for: Electrostatic tuning of the pyridoxal-5′-phosphate cofactor site defines pH dependence in type I cystathionine β-lyases
Source: J Biol Chem. 2026 Apr 17;302(6):111469. doi: 10.1016/j.jbc.2026.111469 (PMC13197776; doi:10.1016/j.jbc.2026.111469)
Supplement: Supporting Information [file mmc1.docx]

**Electrostatic tuning of the pyridoxal-5′-phosphate cofactor site defines pH dependence in type I cystathionine β-lyases**

Yuanxiang Liu^1^, Xin Li^1^, Jianxun Li^1^, Jinyan Liu^1^, Yishu Peng^1^, Yan Gao^1^,Ye Zhang^1^, Zijian Jia^1^, Kexin Kang^1^, Anwei Yin^1^, Cuiqing Ma^1^, Yuechao Yang^2,3^, Chunyu Yang^1^*

1. State Key Laboratory of Microbial Technology, Institute of Microbial Technology,

Shandong University, Qingdao, P. R. China

2. College of Resources and Environment, Shandong Agricultural University, Taian 271018, China.

3. Department of Soil and Water Sciences, Tropical Research and Education Center, IFAS, University of Florida, Homestead, Florida 33031, USA.

*Corresponding author: Prof. Chunyu Yang

Email: ycy21th@sdu.edu.cn

Telephone: 86-0532-58631501

Fax: 86-0532-58631501

**Experimental section**

**Preparation and determination of racemic substrates**

Substrate preparation was modified according to the method described by Adnan Zahirović et al (43). Taking ±S-allyl-l-cysteine sulfoxide (l-(±)-alliin) as an example, the first step for ±S-allyl-l-cysteine preparation is as follows: Mixed 200 mL of ethanol with 40 mL of deionized water, added 0.33 M NaOH, stirring until fully dissolved, and then added 0.165 M L-cysteine for complete dissolution. After adding 0.165 M allyl bromide into the solution, the pH was adjusted to 5.5 using acetic acid, and the resulting white crystals were filtered and dried at 45 °C to obtain ±S-allyl-L-cysteine.

The second step for l-(±)-alliin preparation is as follows: 2 g of ±S-allyl-l-cysteine was first oxidized by adding 2.5 mL of 30% hydrogen peroxide After oxidation, 100 mL of pre-cooled ethanol is added, yielding a white precipitate of L-(±)-alliin. The prepared substrates were characterized by LC-MS using an ESI positive ion source, using column DB-5 ms (30 m × 0.25 mm), under conditions of column temperature range of 50-250 ℃ (5 min), heating rate of 20 ℃ min^-1^, and the injection temperature of 250 ℃. The EI ion source temperature was 250 °C, the electron energy was 70 eV, and the scan range was 20 to 600 m/z.

For ¹H NMR analysis of the synthesized l-(±)-alliin, 5–10 mg of the sample was dissolved in 0.6 mL D₂O and transferred to a 5 mm NMR tube. Spectra were recorded on a 400–600 MHz spectrometer using a water-suppression pulse sequence. The free induction decay (FID) data was processed with MestReNova software (version 10.1). Phase and baseline corrections were applied, the spectrum was referenced to TSP/DSS, and the signals of interest were integrated (44).

**Organic acids assay**

For organic acids assays, all samples were centrifuged at 13,400 × g for 10 min and the supernatant was filtered through a 0.22 μm filter. The samples were measured on the Shimadzu HPLC system (LC 20 AT) equipped with a UV detector and a C18 column (VP ODS 250*4.6/5µm). The mobile phase was 1% trifluoroacetic acid and 20% acetonitrile in water, with a flow rate of 0.5 mL min^-1^, and the column temperature of 40 °C (45).

**Free PLP and enzyme-bound PLP assays**

Following the protocol of previously methods (46, 47), the PLP standard curve was established using PLP solutions at concentrations of 10, 62.5, 100, 125, 250, and 500 μM in 50 mM Tris-HCl buffer (pH 8.0). Absorbance was measured at 400 nm (ΔA_400nm_).

To quantify enzyme-bound PLP, the target proteins were purified by Ni-NTA chromatography and exchanged into Tris-HCl buffer (pH 8.0). Absorbance at 400 nm was measured for each protein sample. A crude lysate (5 mg mL⁻¹ total protein) prepared from *E. coli* BL21(DE3) carrying the empty pET24a vector was used as the background control, and its A_400nm_ value was subtracted (set to zero). All purified protein samples were then diluted to 5 mg mL⁻¹ and measured for the ΔA_400nm_ values. The PLP content was subsequently calculated using the PLP standard curve.

**Supplementary Figures 1-20**

**
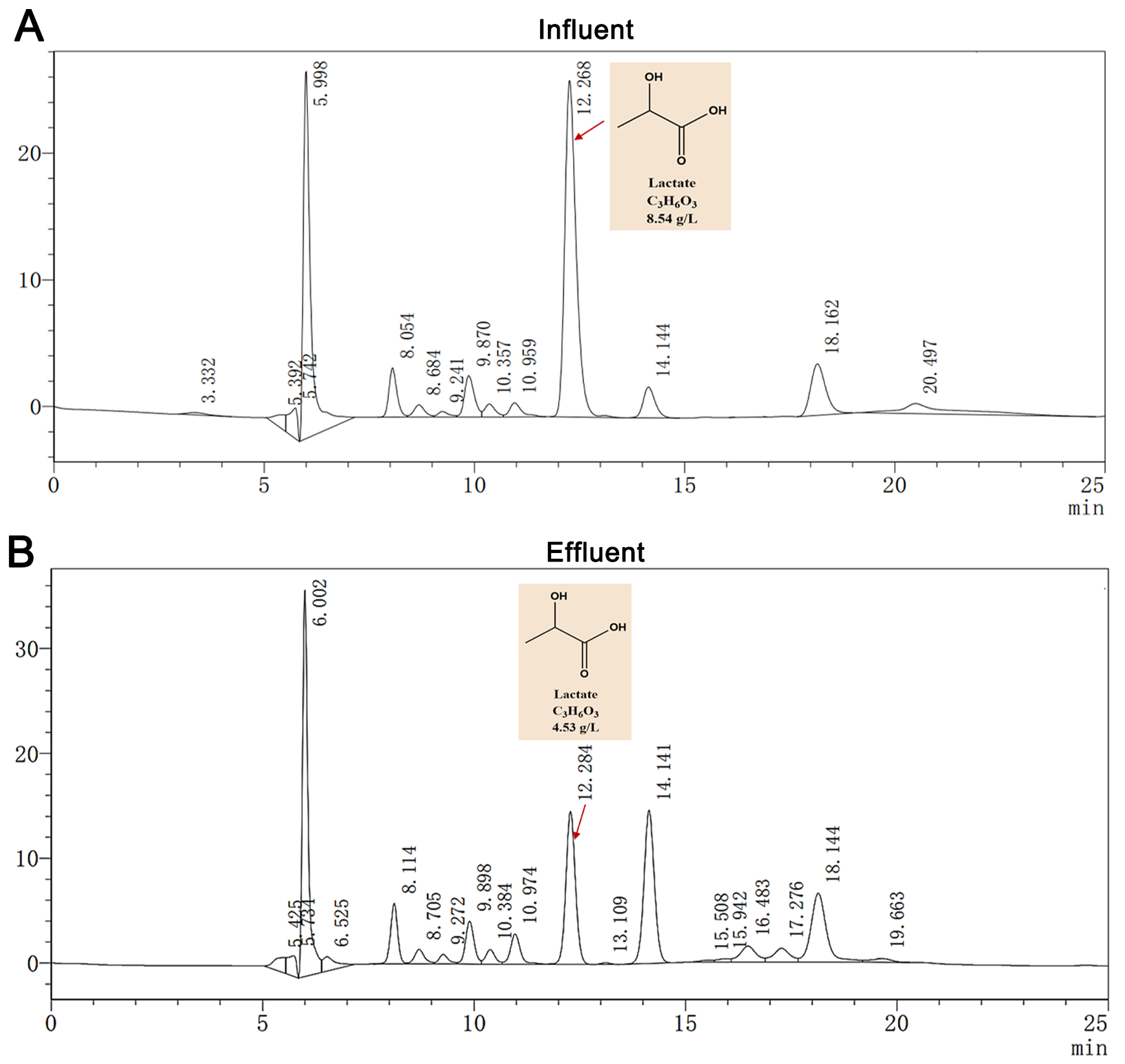
**

**Figure S1. HPLC spectra for organic acid detection in the soybean whey wastewater (SWW).** (**A**) SWW influent. (**B**) SWW effluent.

**
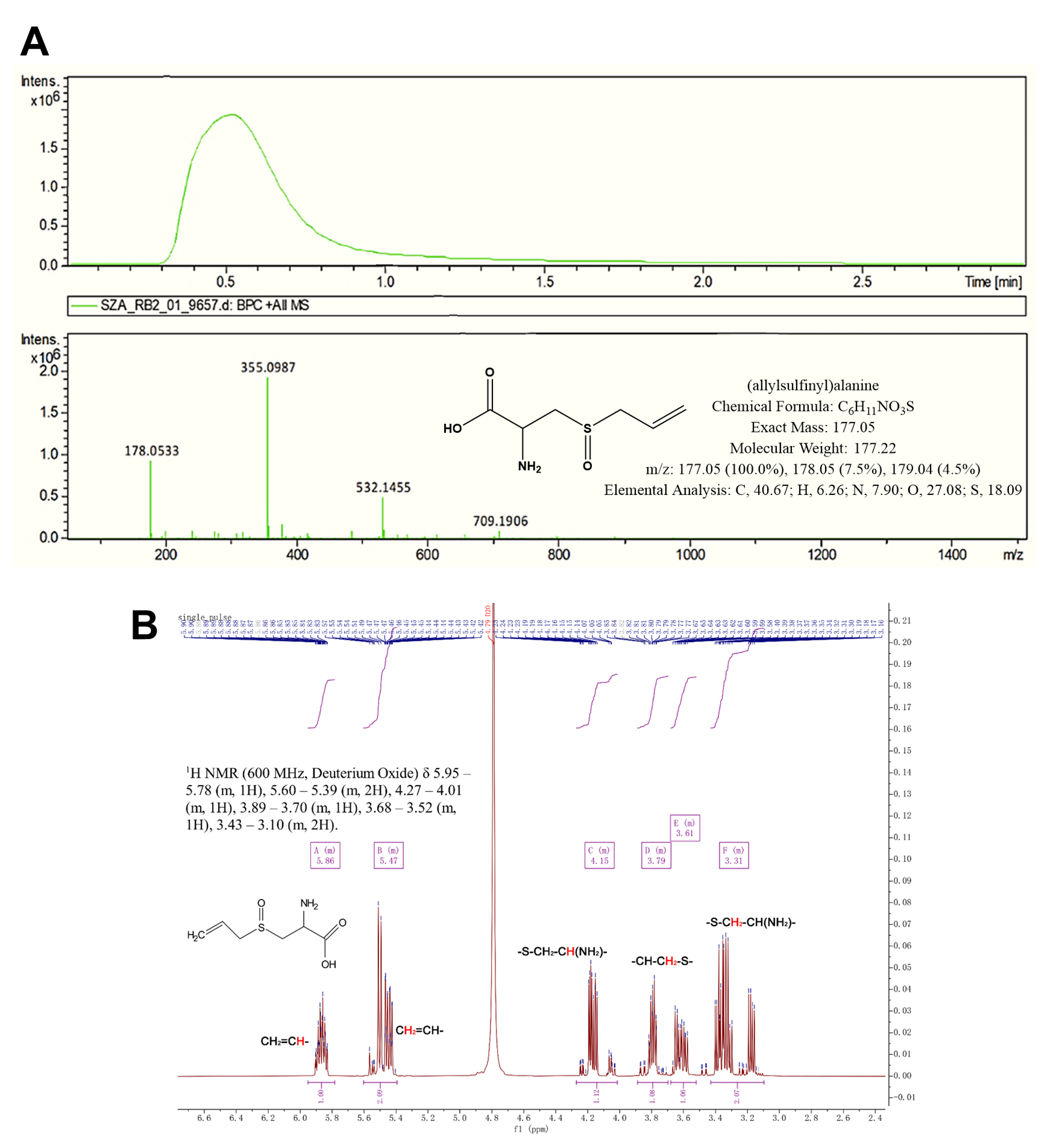
**

**Figure S2.** LC-MS and ^1^H NMR spectra of chemically synthesized l-(±)-alliin. (A) HPLC-MS detection of ±S-allyl-l-cysteine sulfoxide (l-(±)-alliin, C_6_H_11_NO_3_S, M_W_ 177.22). (B) ^1^H NMR detection of ±S-allyl-l-cysteine sulfoxide.


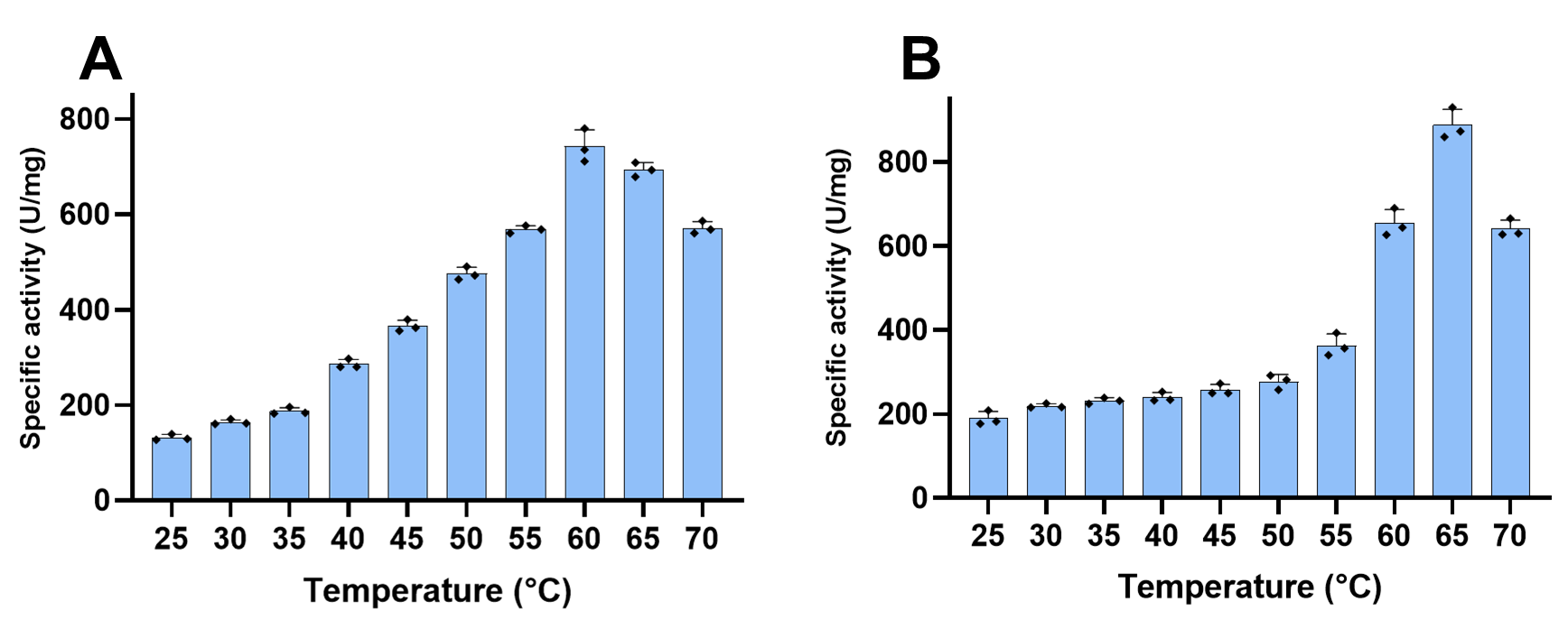


**Figure S3. Optimal reaction temperatures of LdPatB (A) and MePatB (B).** The specific activity (U mg^-1^) was measured at various temperatures ranging from 25 °C to 70 °C. Error bars represent standard deviation from three independent experiments.


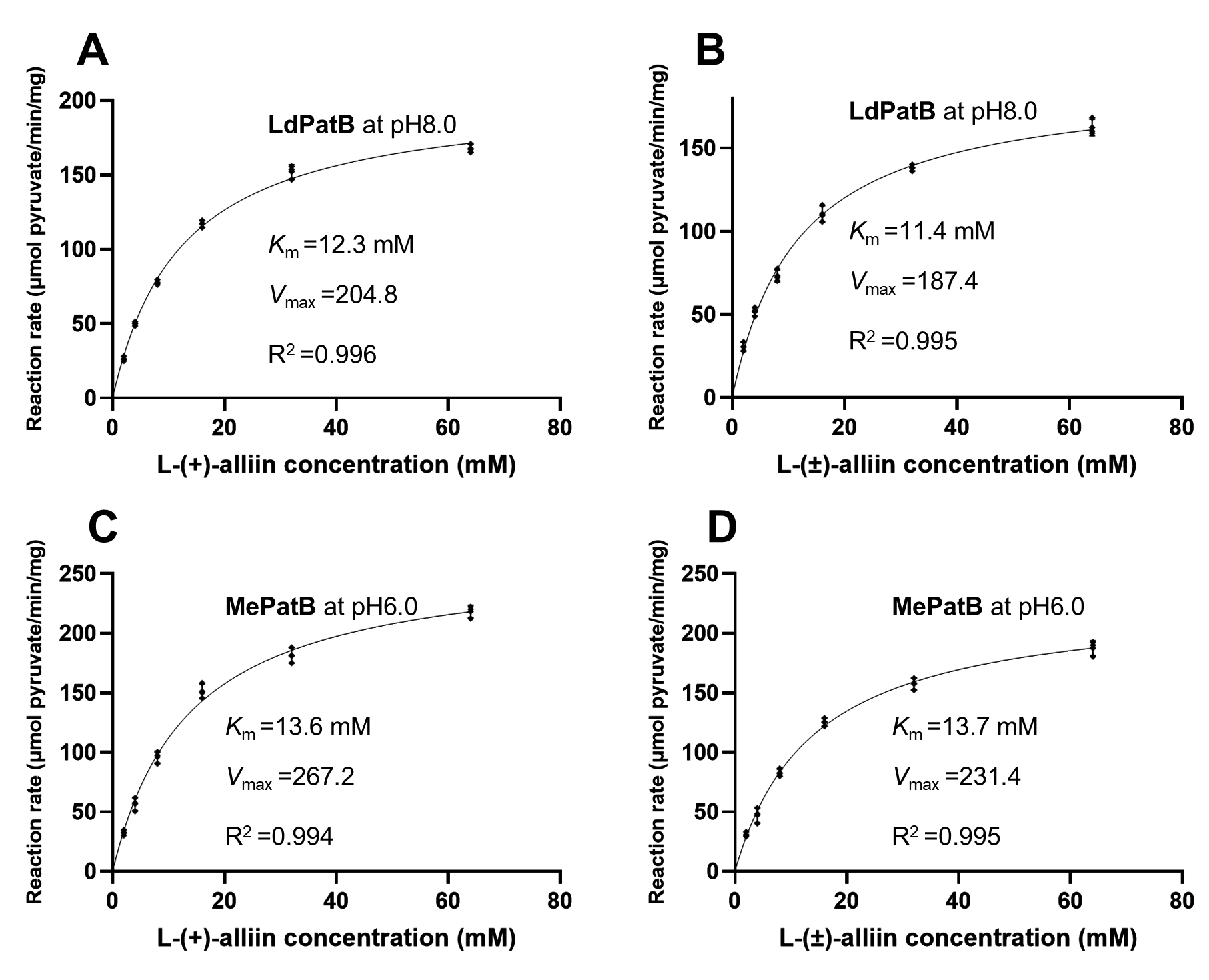


**Figure S4. Michaelis-Menten kinetic curves of LdPatB and MePatB with l-(±)-alliin and l-(+)-alliin at their respective optimal pH of 8.0 and 6.0** (A) Non-linear fitting curve of *K*_m_ and *V*_max_ for LdPatB with l-(+)-alliin as the substrate. (B) Non-linear fitting curve of *K*_m_ and *V*_max_ for LdPatB with l-(±)-alliin as the substrate. (C) Non-linear fitting curve of *K*_m_ and *V*_max_ for MePatB with l-(+)-alliin as the substrate. (D) Non-linear fitting curve of *K*_m_ and *V*_max_ for MePatB with l-(±)-alliin as the substrate. The final concentration of the purified enzyme used was 0.05 mg/mL. All experiments were performed in triplicate.

**
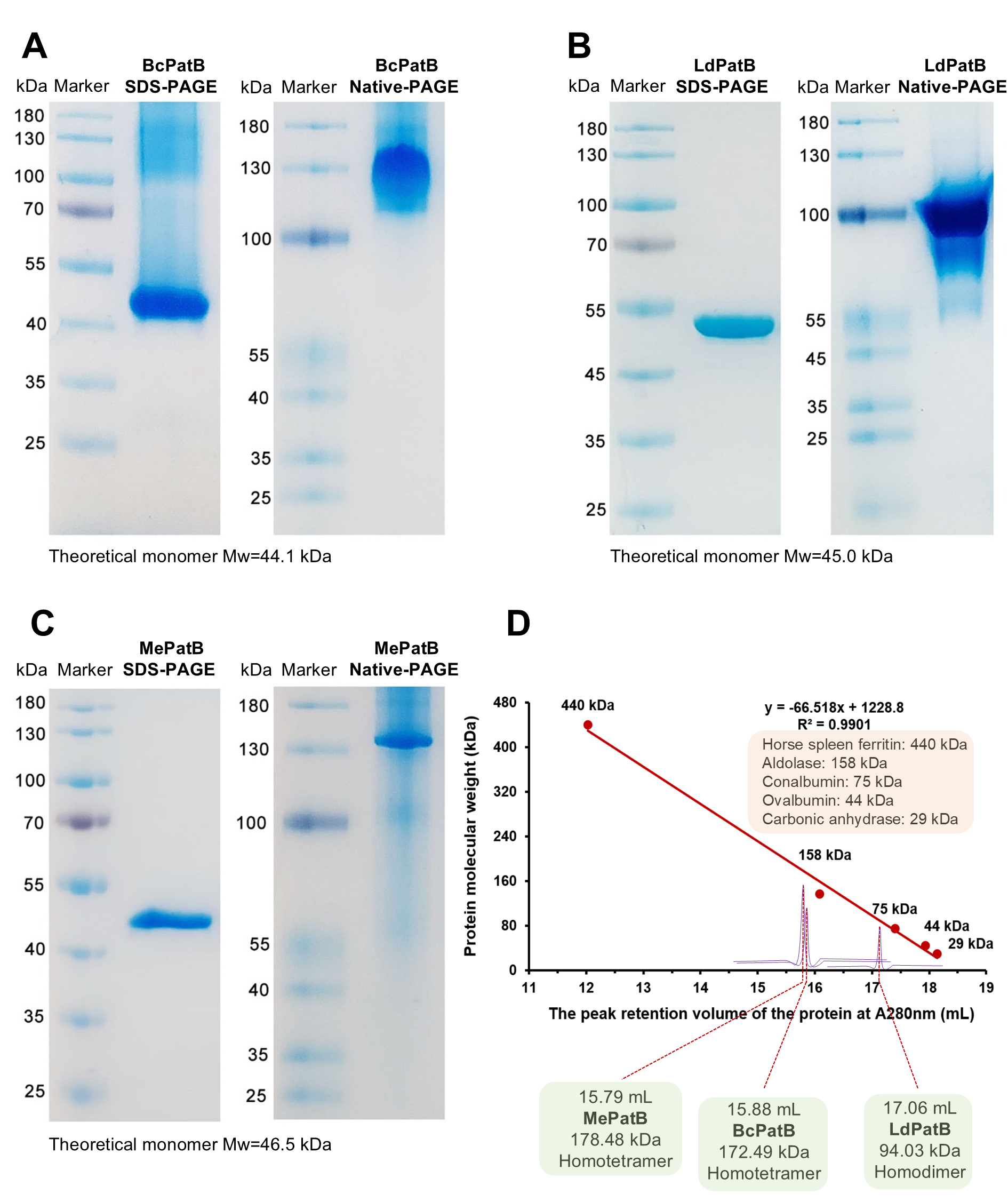
**

**Figure S5. SDS-PAGE and size-exclusion chromatography (SEC) analyses of purified BcPatB, LdPatB, and MePatB.** (A–C) SDS-PAGE and Native-PAGE spectra of Ni-NTA-purified BcPatB, LdPatB, and MePatB. No reducing agent was included in the Native-PAGE. BcPatB migrates as a single band at approximately 45 kDa (theoretical monomer mass 44.1 kDa). LdPatB shows a single band around 46 kDa (theoretical monomer mass 45.0 kDa). MePatB displays a single band around 46 kDa (theoretical monomer mass 46.5 kDa). (D) SEC analysis of BcPatB, LdPatB, and MePatB. The elution profile was monitored at A_280nm_, and the protein molecular weight was estimated using a calibration curve derived from standard proteins (horse spleen ferritin: 440 kDa; aldolase: 158 kDa; conalbumin: 75 kDa; ovalbumin: 44 kDa; carbonic anhydrase: 29 kDa). The calibration curve is shown as Y = -66.518x + 1228.8 (*R*² = 0.9901). The retention volumes and calculated molecular weights indicate that BcPatB and MePatB exist as homotetramers (172–178 kDa), while LdPatB forms a homodimer (94 kDa). The theoretical molecular weight (Mw) of the monomeric protein was calculated using the ProtParam tool on the ExPASy server.


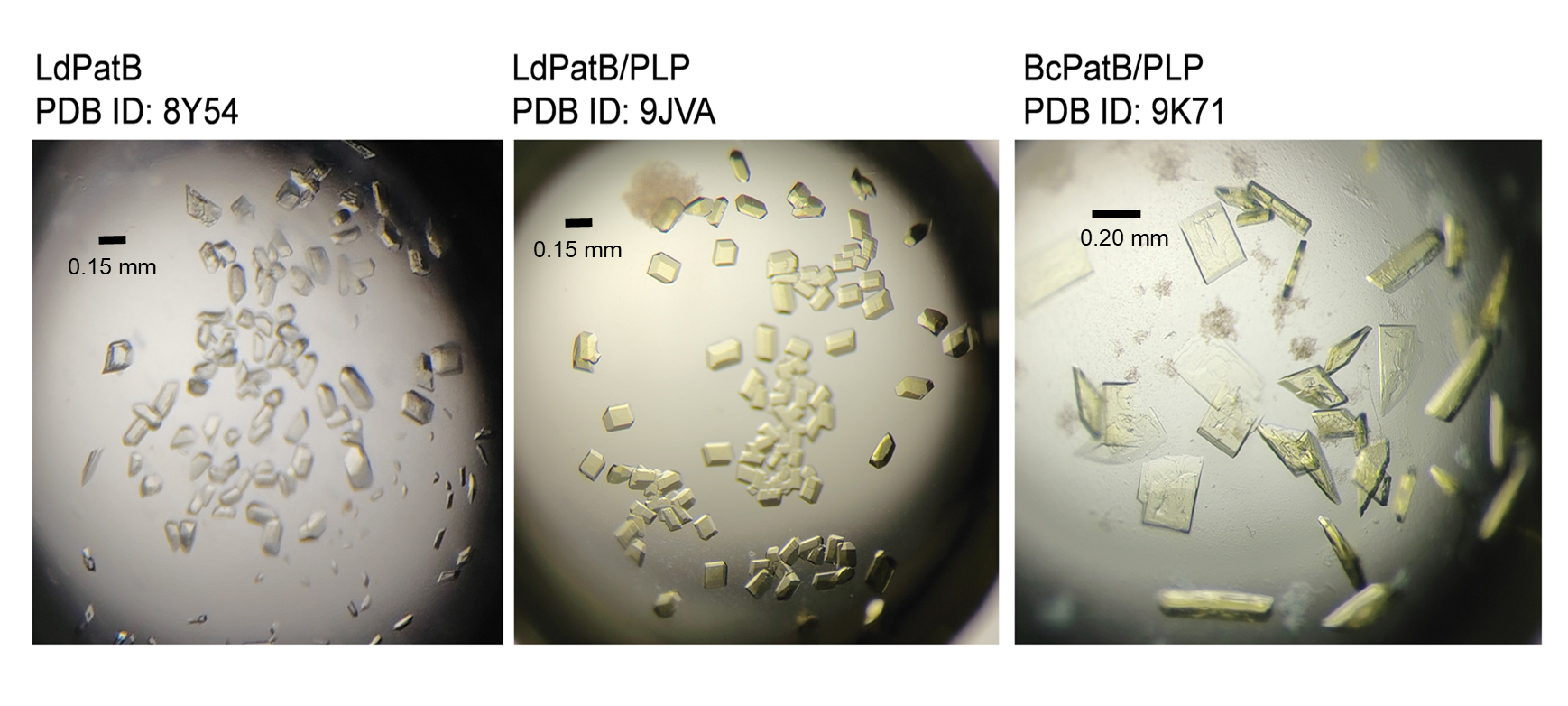


**Figure S6. Microscopic images of protein crystals obtained for LdPatB, LdPatB/PLP, and BcPatB/PLP complexes.** Crystals were visualized under a light microscope, and scale bars are indicated in each panel (0.15 mm for LdPatB and LdPatB/PLP; 0.20 mm for BcPatB/PLP).

**
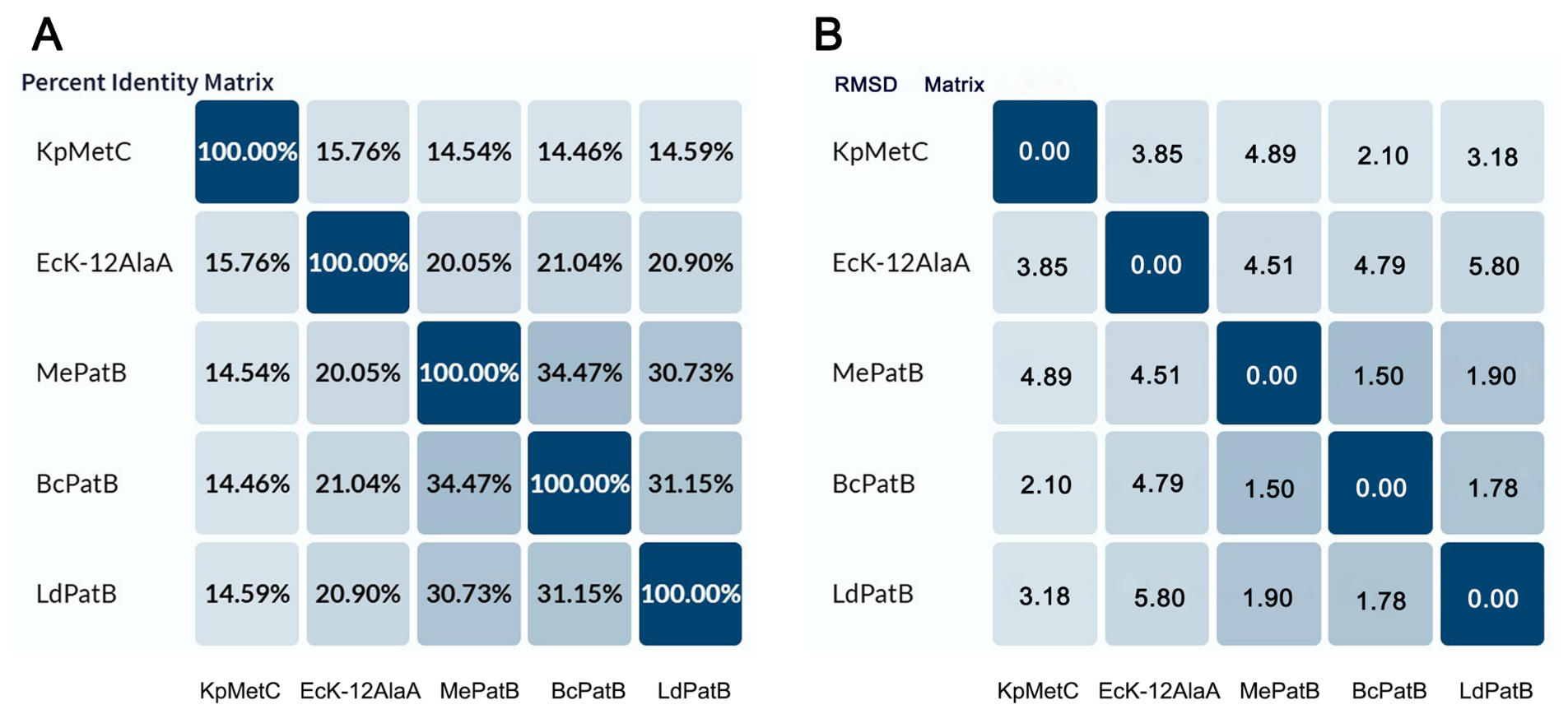
**

**Figure S7. Amino acid sequence percent identity matrix (A) and RMSD (root mean square deviation) Matrix (B) among KpMetC, Eck-12AlaA, MePatB, BcPatB, and LdPatB.** The RMSD (Å) matrix was calculated from the superposition of the three-dimensional structures of the five proteins.

**
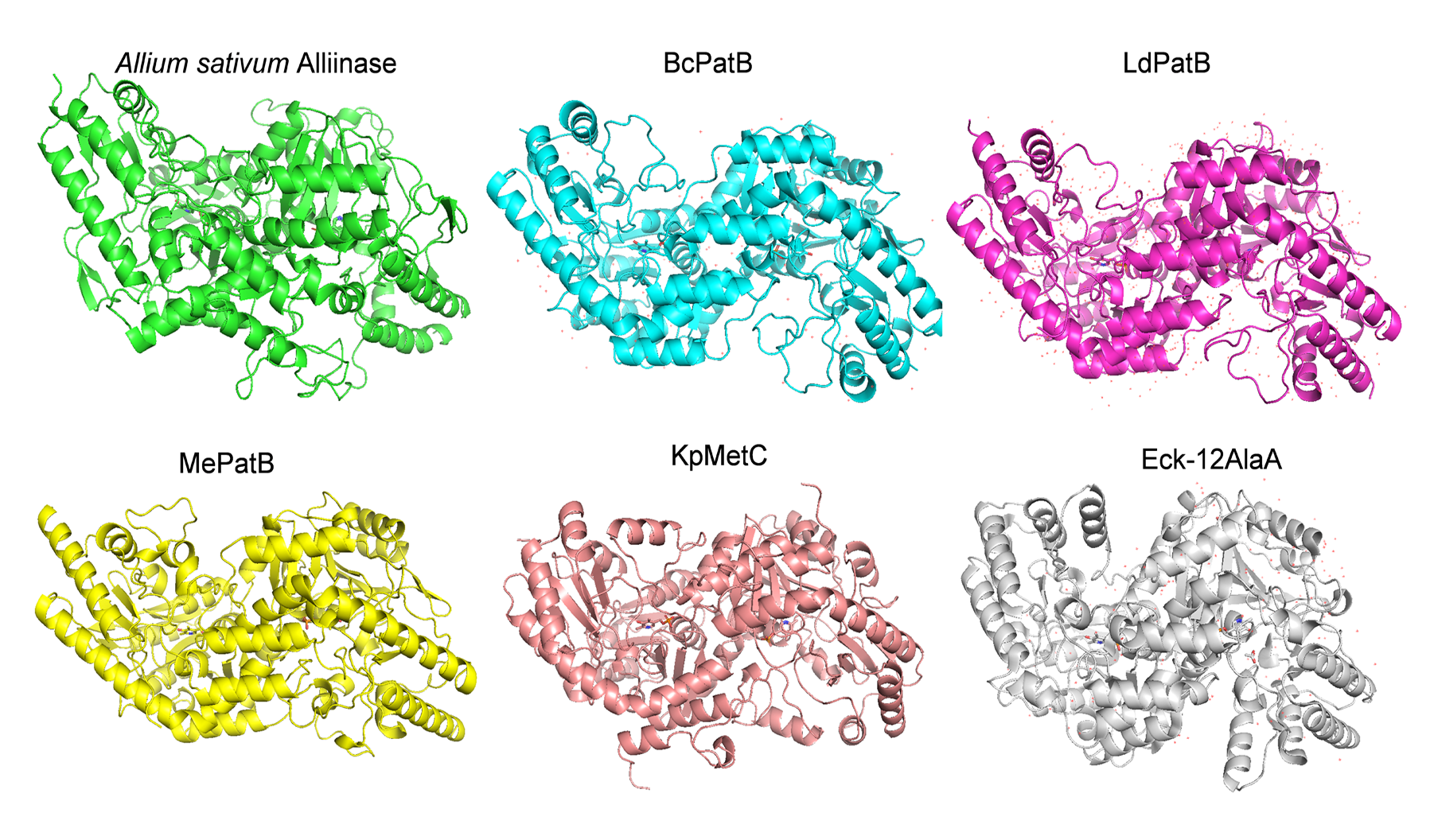
**

**Figure S8. Three-dimensional structural models of several PLP-dependent enzymes with different folding patterns**. *Allium sativum* alliinase is shown in green and is derived from PDB entry 1LK9 (22). BcPatB with PLP ligand is depicted in cyan and was resolved in this study at pH 7.0 (PDB ID: 9K71). LdPatB is illustrated in magenta and was also resolved in this study (PDB ID: 8Y54). MePatB is presented in yellow and was modeled using AlphaFold3. KpMetC is shown in pink and was modeled using AlphaFold3. Eck-12AlaA is represented in gray and is derived from PDB entry 4CVQ (27). Visualizations were performed using PyMOL.

**
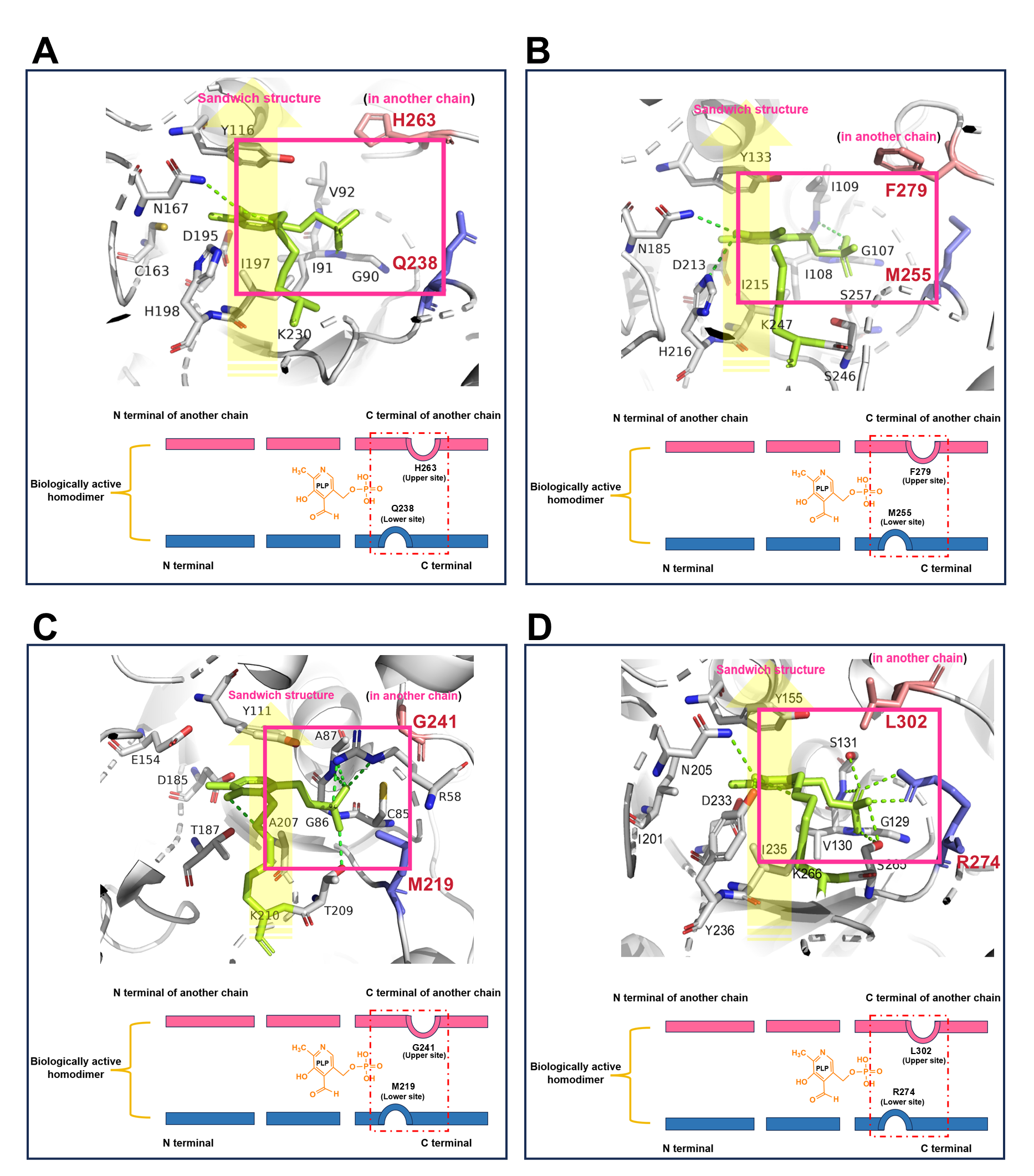
**

**Figure S9. 3D and two-dimensional representations of the structurally conserved residue pair.** The structurally conserved residue pair is indicated in bold and red. The composition and simplified 2-D diagrams of the residue pairs are shown for cystathionine β-lyase BcPatB (A), MePatB (B), KpMetC (C), and alanine aminotransferase Eck-AlaA (D).

**
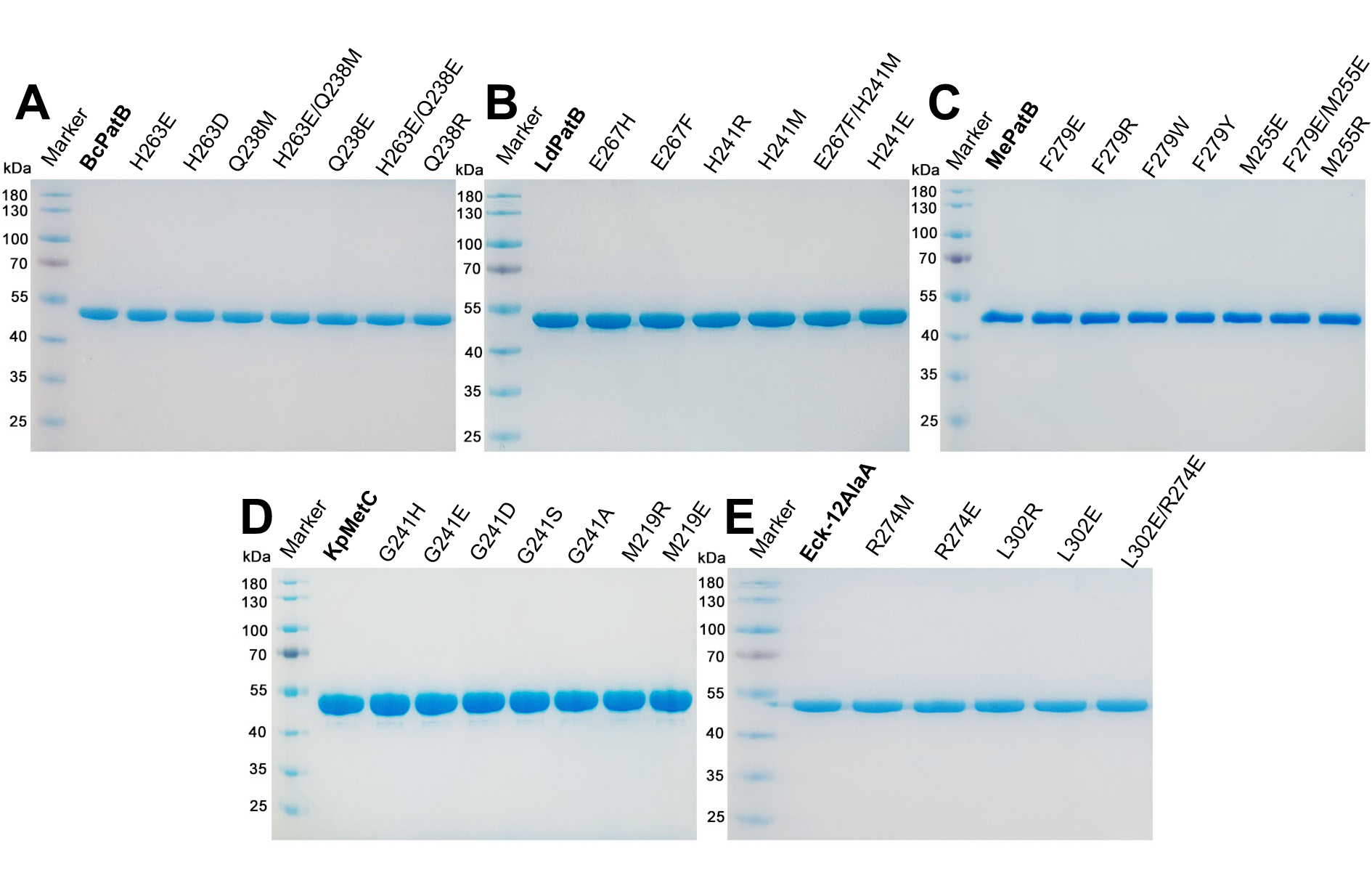
**

**Figure S10.** **SDS-PAGE spectra of the purified wild-type enzymes and their mutants.** Protein samples were separated by 10% SDS-PAGE and visualized using Coomassie Brilliant Blue staining. Molecular weight markers (kDa) are shown on the left side of each panel. (A) SDS-PAGE image of Ni-NTA purified BcPatB and its variants (H263E, H263D, Q238M, H263E/Q238M, Q238E, H263E/Q238R, and Q238R). (B) SDS-PAGE image of LdPatB and its variants (E267H, E267F, H241R, H241M, E267F/H241M, H241E). (C) SDS-PAGE image of MePatB and its variants (F279E, F279R, F279W, F279Y, M255E, F279E/M255E, M255R). (D) SDS-PAGE image of KpMetC and its variants (G241H, G241E, G241D, G241S, G241A, M219R, M219E). (E) SDS-PAGE image of Eck-12AlaA and its variants (R274M, R274E, L302R, L302E, L302E/R274E). All protein bands appear at approximately 40–55 kDa, indicating successful expression and proper molecular size for all constructs. ImageJ analysis confirmed >95% purity for each protein.

**
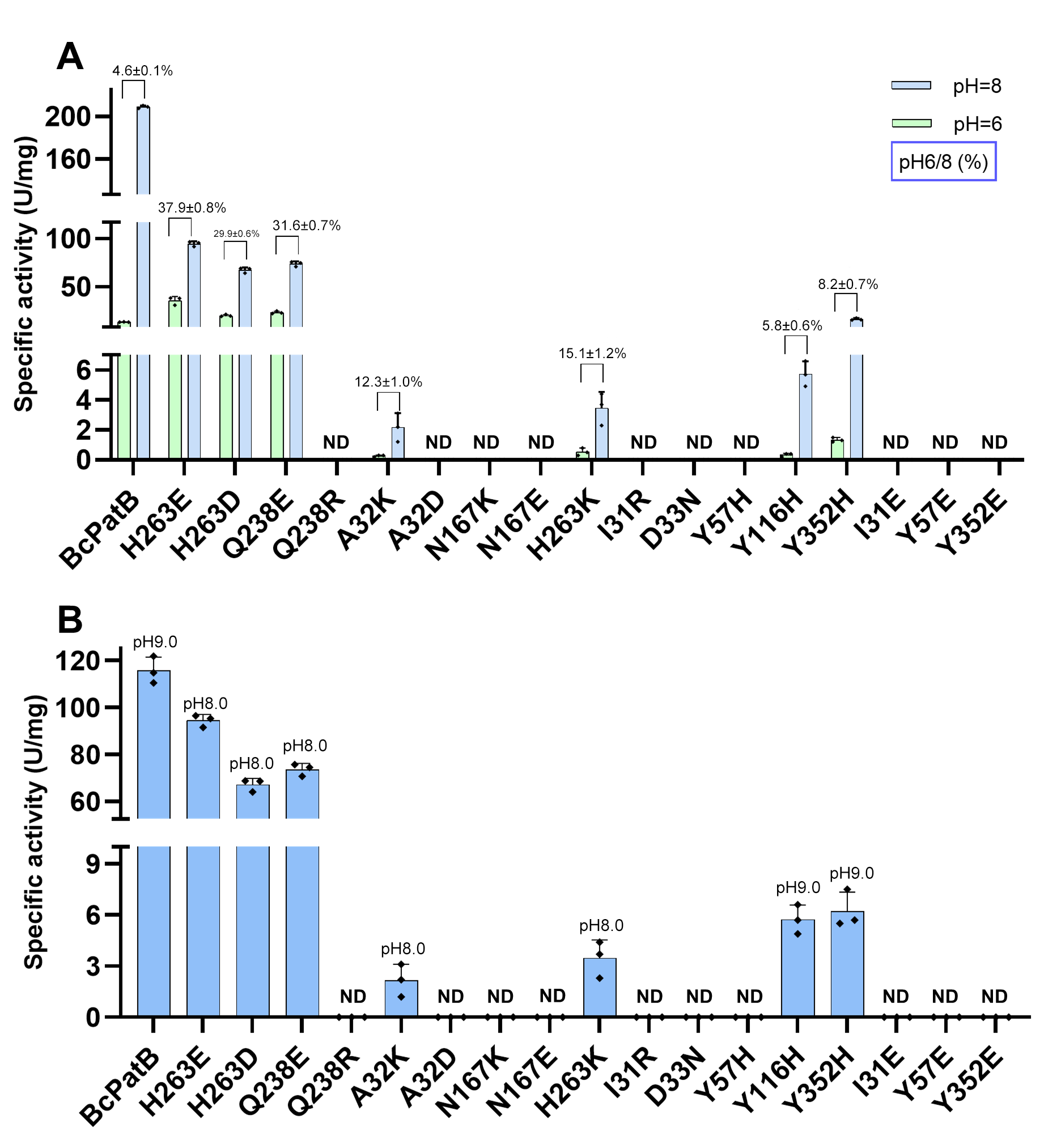
**

**Figure S11. Specific activity and pH6/pH8 ratio values for BcPatB variants**. The relative enzyme activity at pH6/8 (%) is calculated to represent the enzyme’s preference for mildly acidic pH. (A) Bar chart shows the specific activity of various BcPatB variants at pH 6.0 and 8.0, with pH6/8 ratio values indicated above each bar. (B) Bar chart displays the specific enzyme activity of BcPatB variants at their respective optimal pH values, with optimal pH values indicated above each bar. “ND” denotes activity that was not detectable. Error bars represent the standard deviation from three independent experiments.


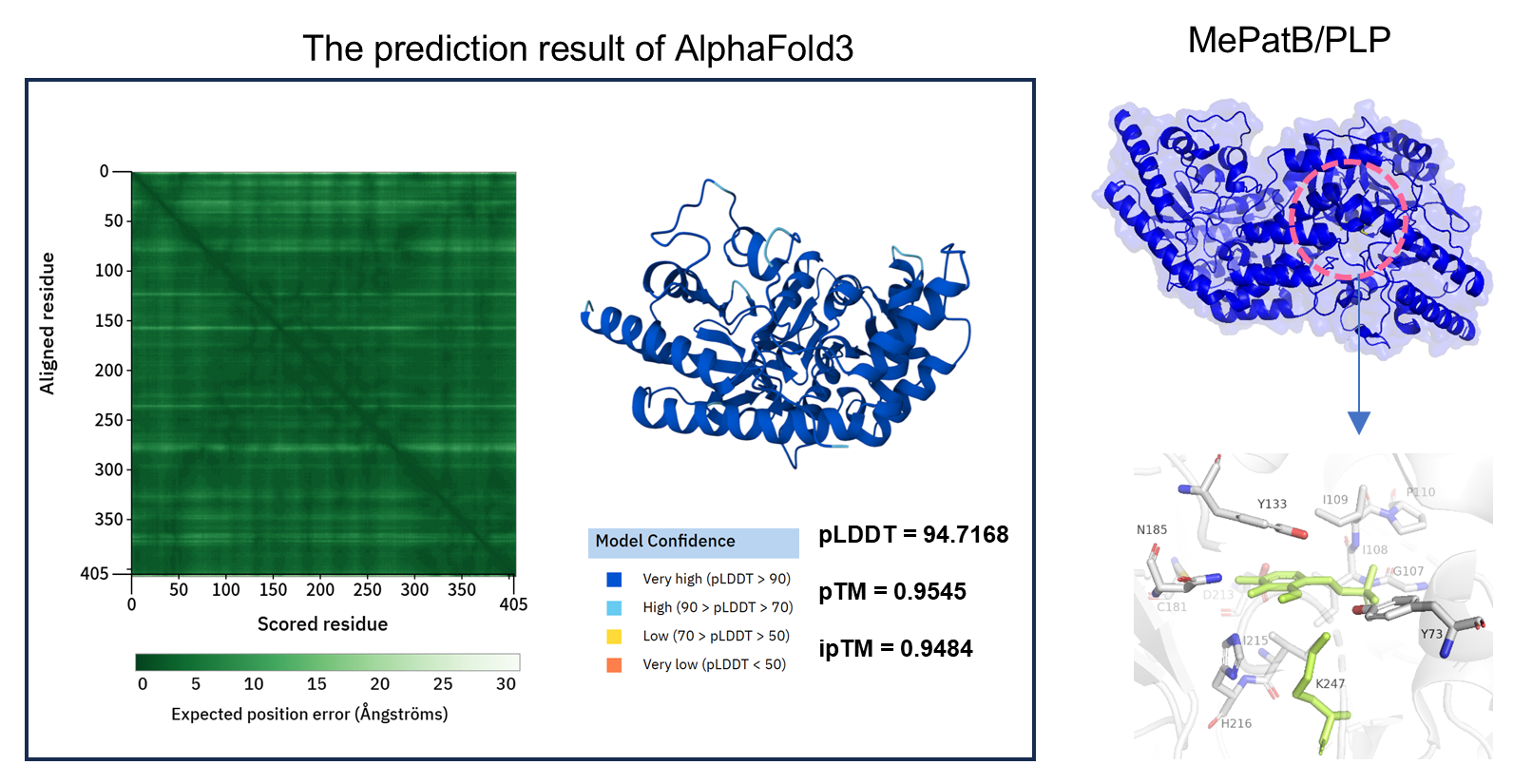


**Figure S12. Cartoon representation and close-up view of MePatB/PLP structure predicted by AlphaFold3.** High pLDDT values (>90) indicate high confidence in local structure prediction, while pTM and ipTM reflect global fold accuracy and inter-domain topology, respectively.

**
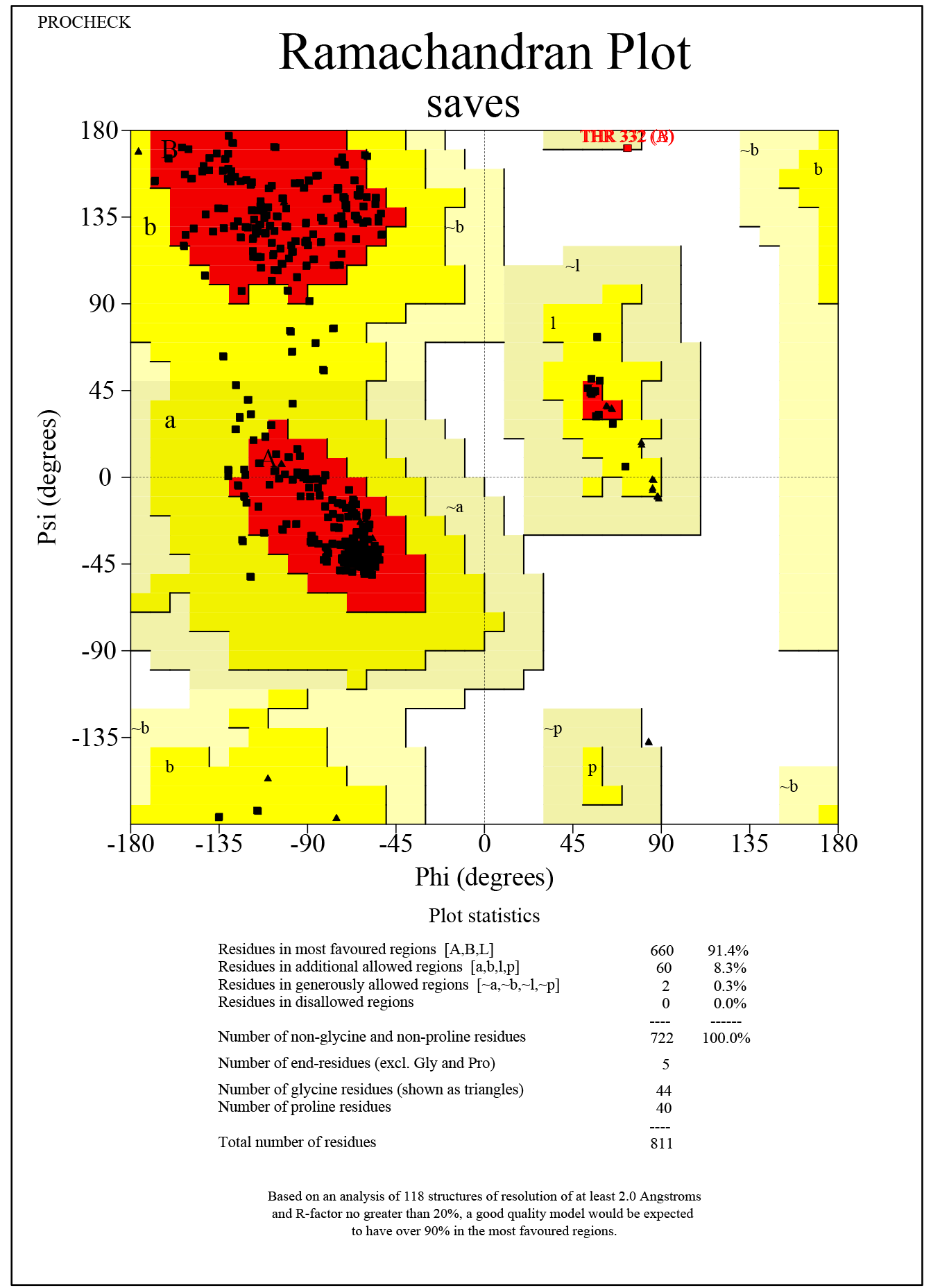
**

**Figure S13. Ramachandran plot for Alphafold3-modeled MePatB/PLP.** The plot provides a visual representation of the phi (Φ) and psi (Ψ) torsion angles for the protein structure of MePatB, which is essential for evaluating the quality of the protein model. A high-quality model is expected to have over 90% of its residues in the most favored regions [A, B, L], which is consistent with the MePatB enzyme model shown in this plot.


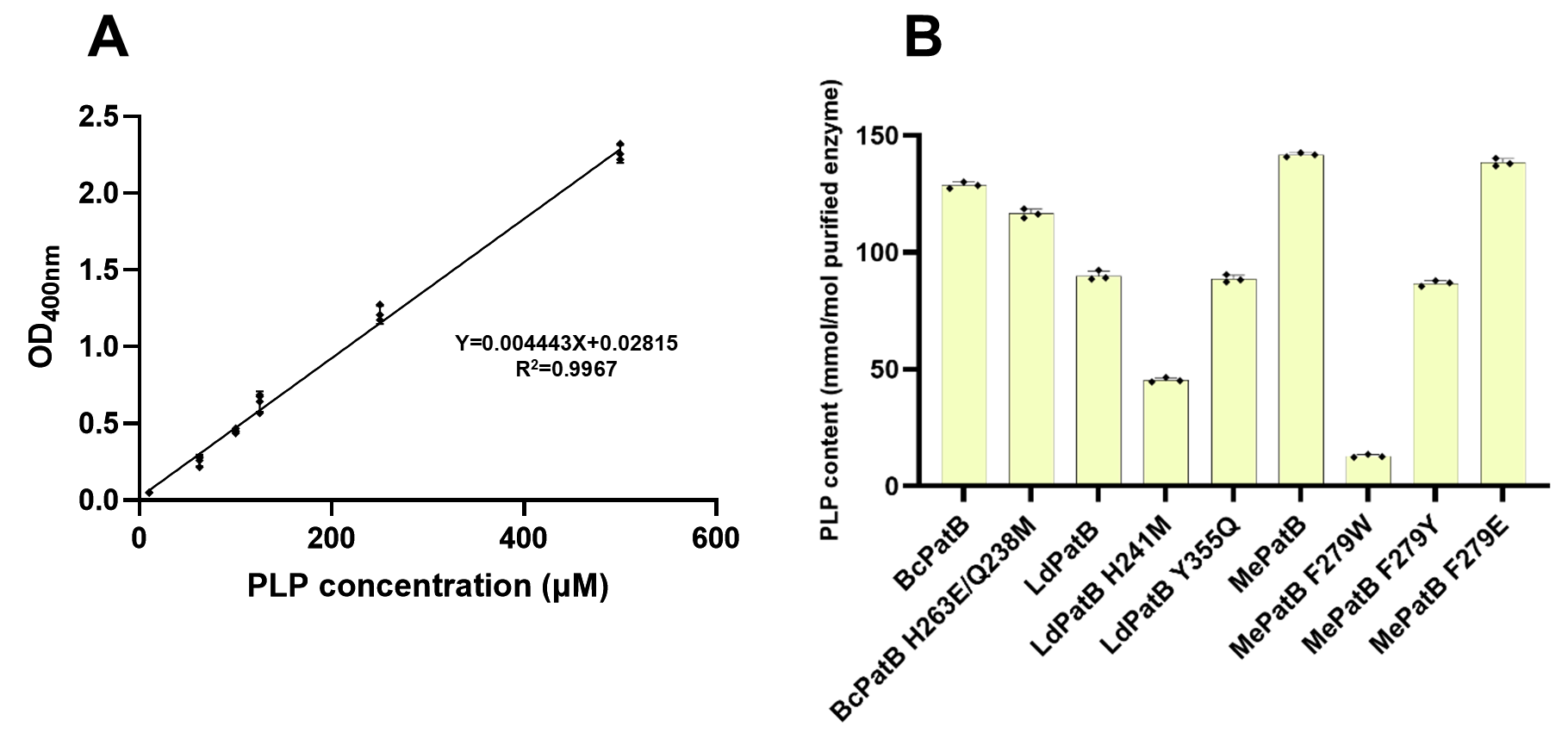


**Figure S14. Standard curve for PLP quantification (A) and PLP-binding concentrations of PatB mutants (B).** Error bars represent the standard deviation from three independent experiments.


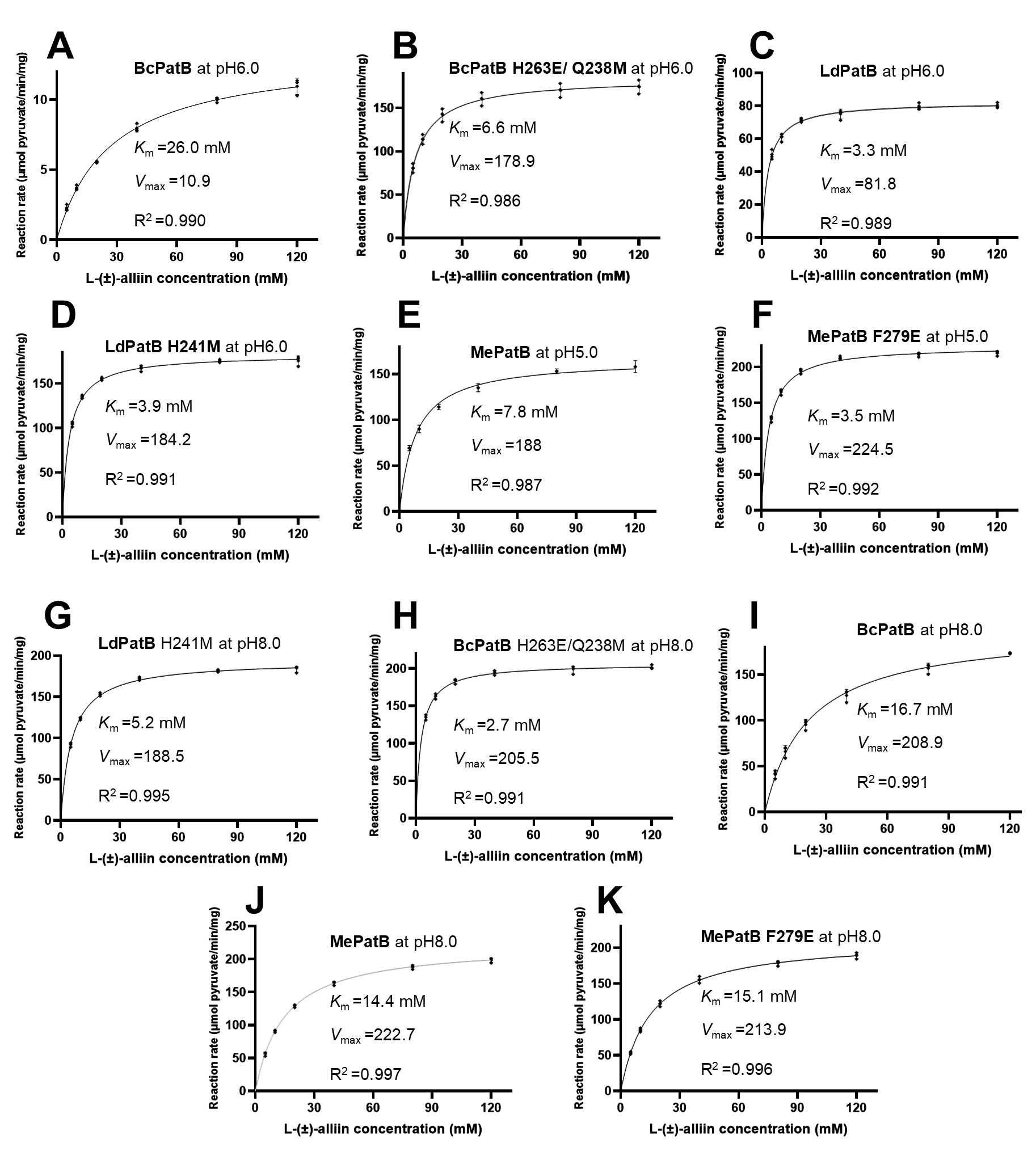


**Figure S15. Kinetic fitting curves of PatB and key mutants toward l-(±)-alliin at 35 °C under different pH values**. (A) BcPatB at pH 6.0. (B) BcPatB H263E/Q238M at pH 6.0. (C) LdPatB at pH 6.0. (D) LdPatB H241M at pH 6.0. (E) MePatB at pH 5.0. (F) MePatB F279E at pH 5.0. (G) LdPatB H241M at pH 8.0. (H) BcPatB H263E/Q238M at pH8.0. (I) BcPatB at pH 8.0. (J) MePatB at pH 8.0. (K) MePatB F279E at pH 8.0. The final concentration of the purified enzyme used in the system was 0.02‒0.1 mg/mL. All experiments were performed in triplicate.


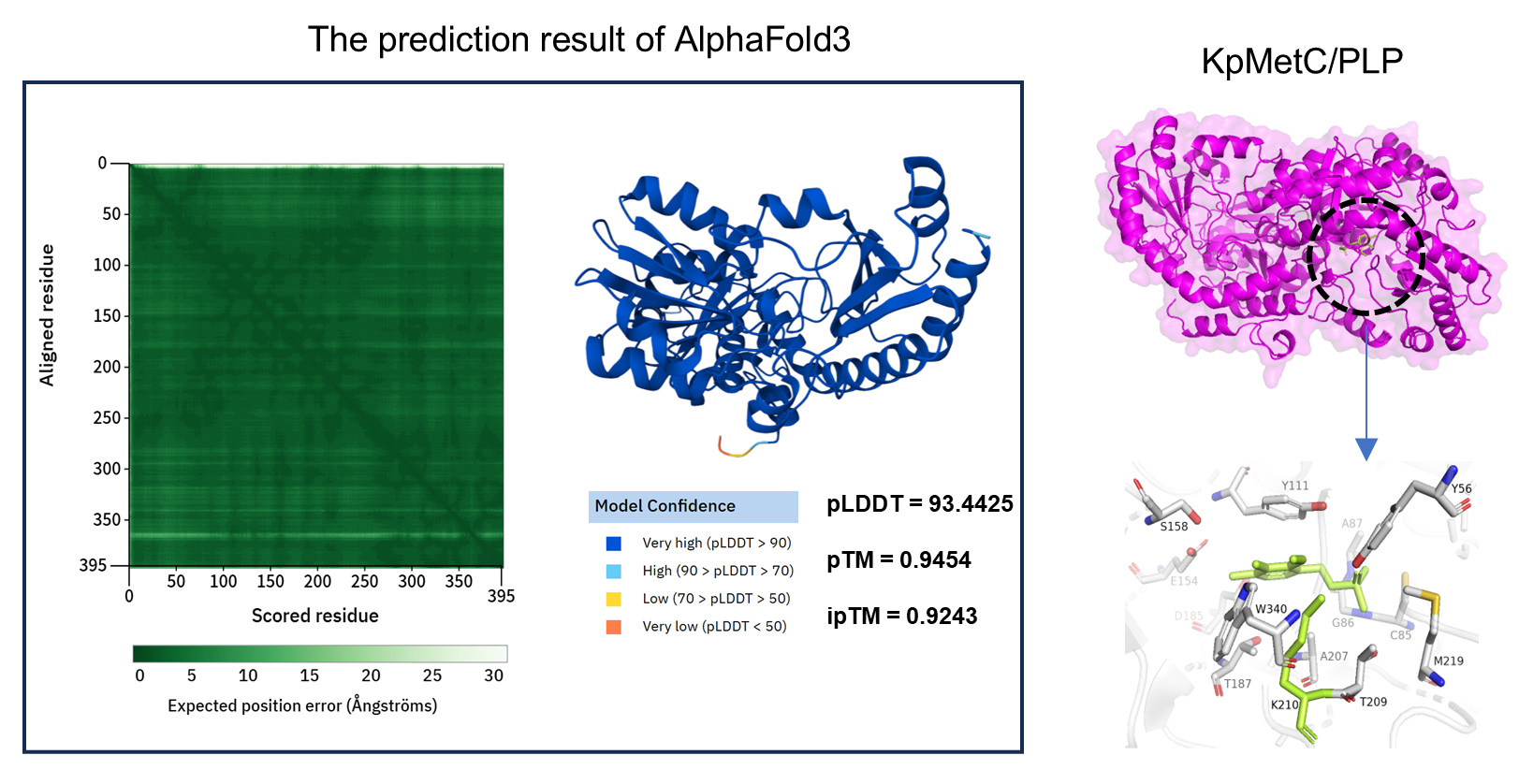


**Figure S16. Cartoon representation and close-up view of KpMetC/PLP structure predicted by AlphaFold3.** High pLDDT values (>90) indicate high confidence in local structure prediction, while pTM and ipTM reflect global fold accuracy and inter-domain topology, respectively.


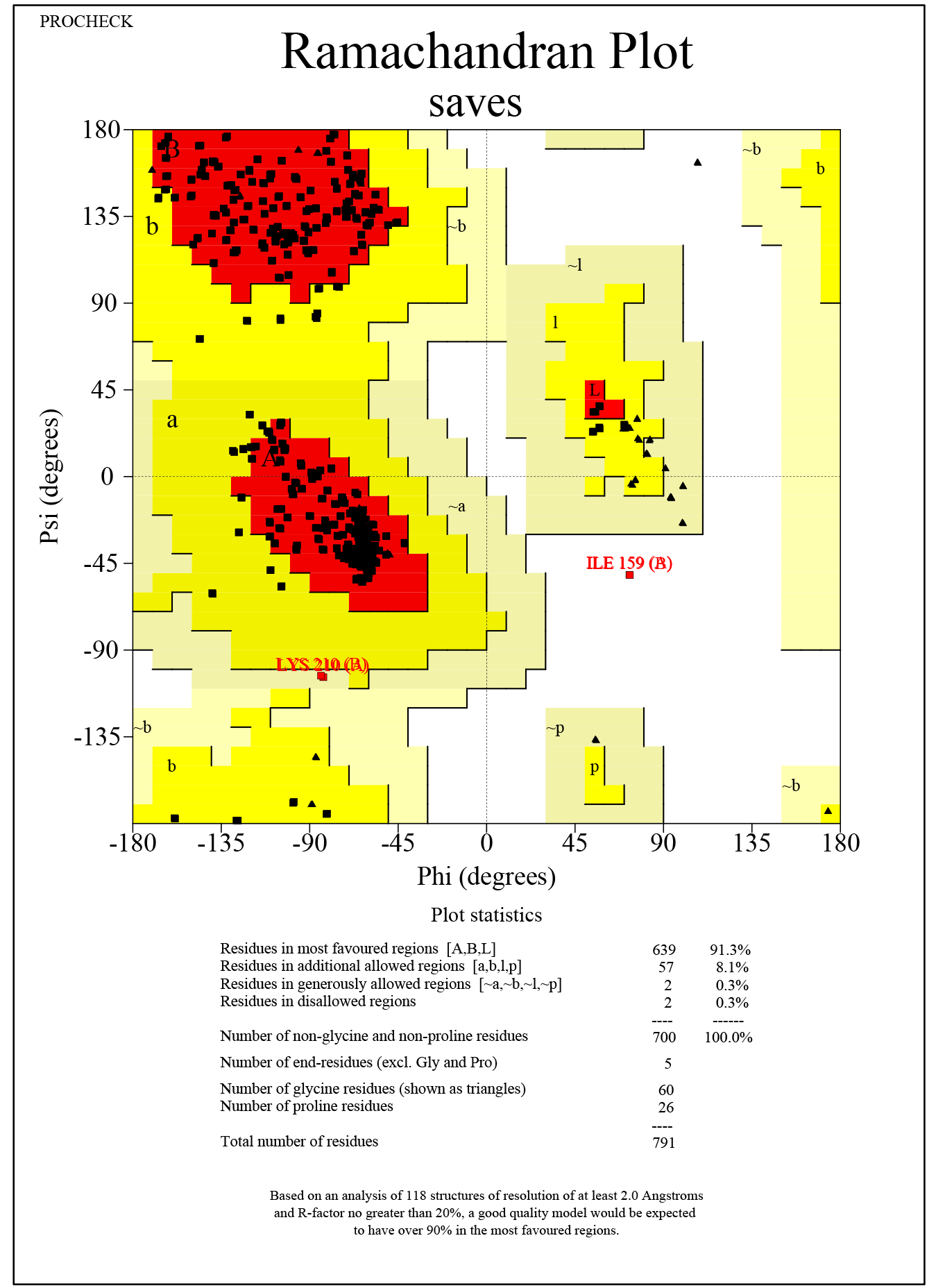


**Figure S17. Ramachandran plot for Alphafold3-modeled KpMetC/PLP.** The plot provides a visual representation of the phi (Φ) and psi (Ψ) torsion angles for the protein structure of KpMetC, which is essential for evaluating the quality of the protein model. A high-quality model is expected to have over 90% of its residues in the most favored regions [A, B, L], which is consistent with the KpMetC enzyme model shown in this plot.

**
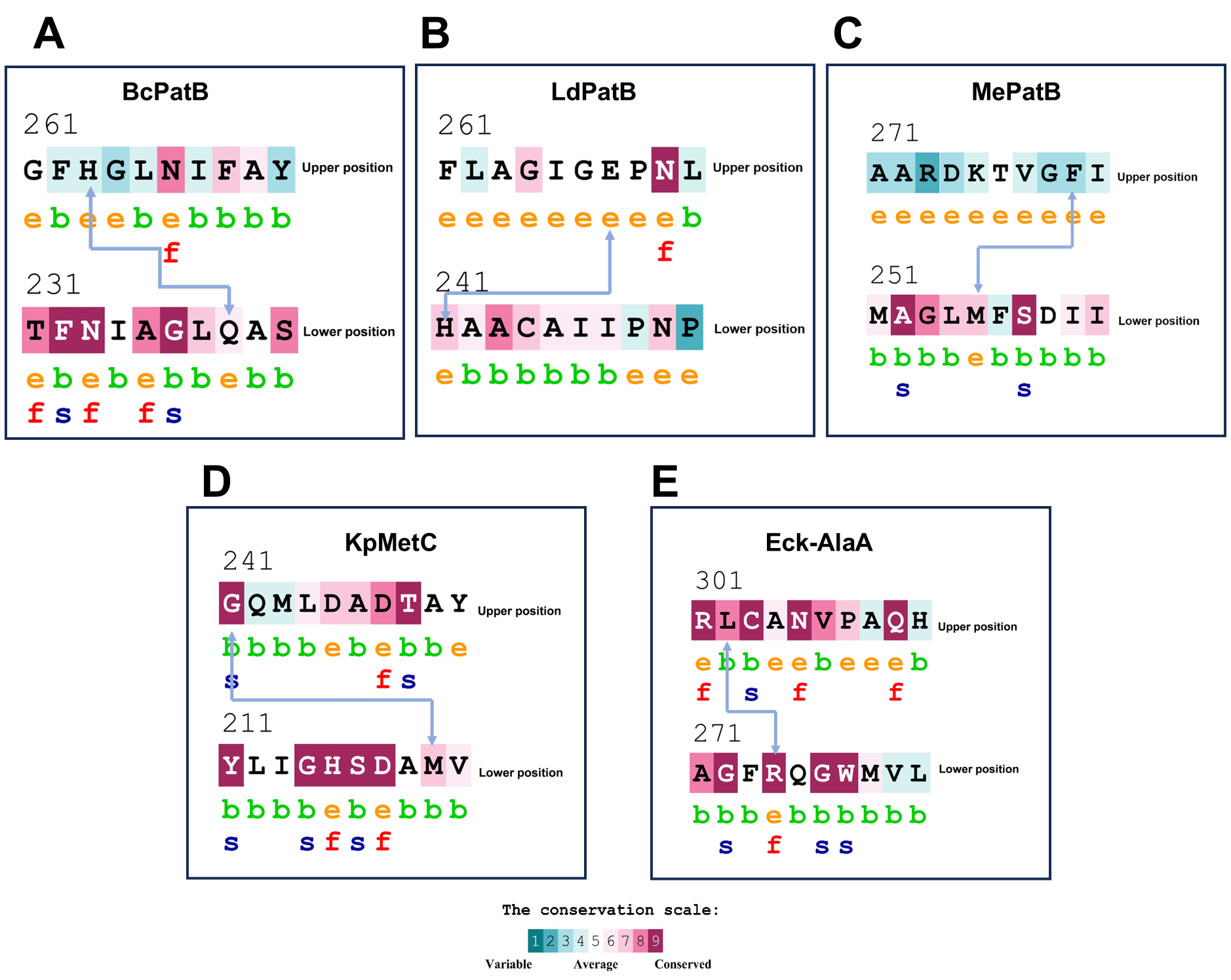
**

**Figure S18. Evolutionary conservation analysis of the “residue pair” in fold-type I PLP-dependent enzymes of BcPatB, LdPatB, MePatB, KpMetC, and Eck-AlaA.** The arrow indicates the residue pair investigated in this study. The analysis was conducted using the ConSurf server with the default HomoloGene pipeline (150 sequences, E-value ≤ 10⁻³, sequence identity 35–95%) (41). Conservation scores are mapped from 1 (variable, cyan) to 9 (absolutely conserved, maroon). The residue pairs in PatBs show relatively lower conservation (average score 3–7), while those in KpMetC and Eck-12AlaA are highly conserved within their respective enzyme sub-families (scores 7–9).


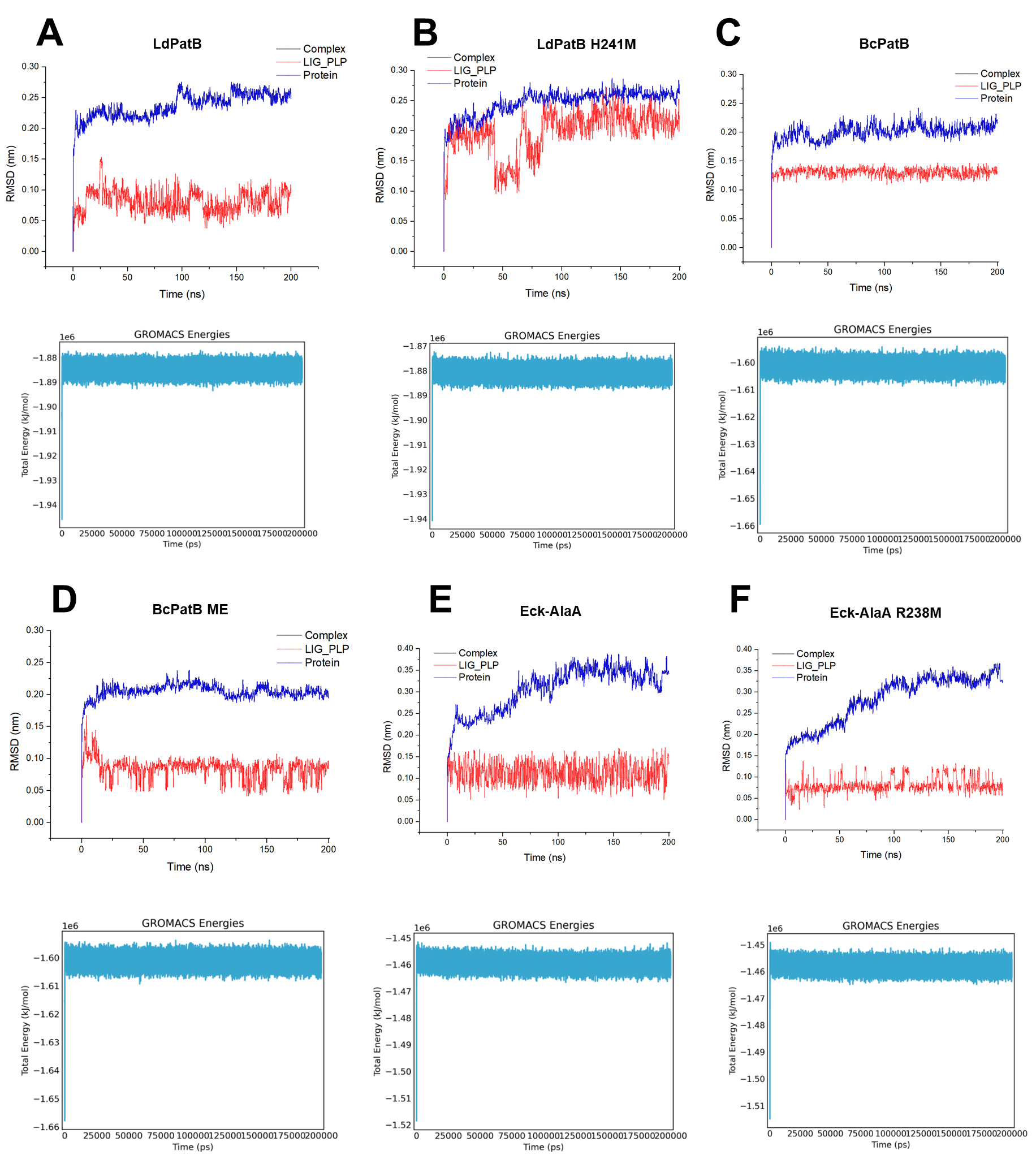


**Figure S19. RMSD profiles and system energy of proteins during Constant pH molecular dynamic simulations at pH 6.0.** The RMSD and energy analyses of the following complexes are shown: (A) LdPatB–PLP, (B) LdPatB H241M–PLP, (C) BcPatB–PLP, (D) BcPatB H263E/Q238M–PLP, (E) Eck-AlaA–PLP, and (F) Eck-AlaA R238M–PLP.


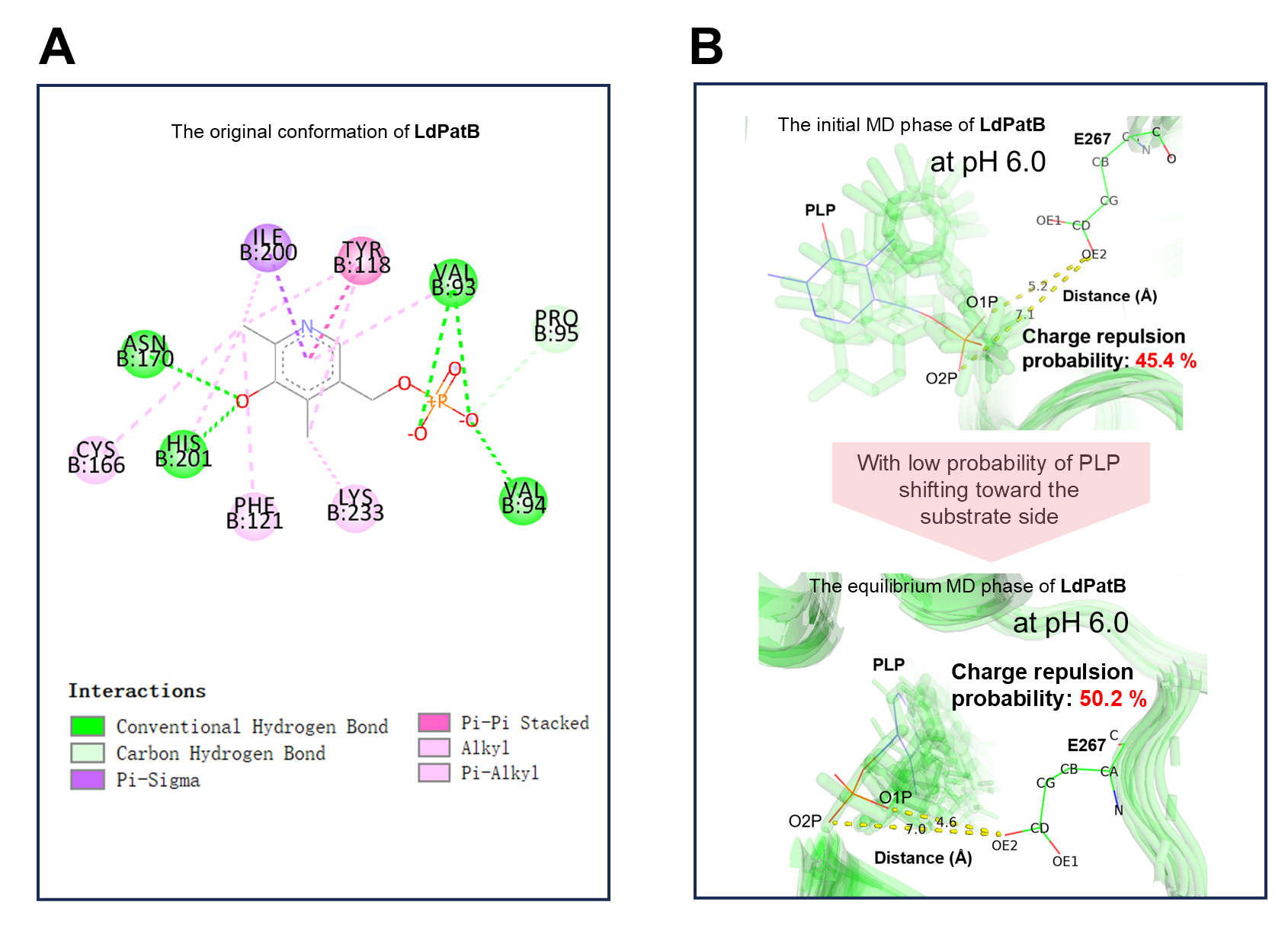


**Figure S20. Constant-pH molecular dynamics simulations for LdPatB at pH 6.0.** (A) The original conformation (frame 0) of LdPaB. (B) The initial (frames 0-10, upper panel) and equilibrium MD phase (frames 175-200, lower panel) of LdPatB. The upper panel shows the distance between PLP and E267 in the initial phase, with a 45.4% probability of forming charge repulsion interactions; the lower panel shows the distance and a 50.2% probability of forming charge repulsion in the equilibrium phase. The distances between atoms of PLP and the residue pair are indicated by yellow dashed line.

**
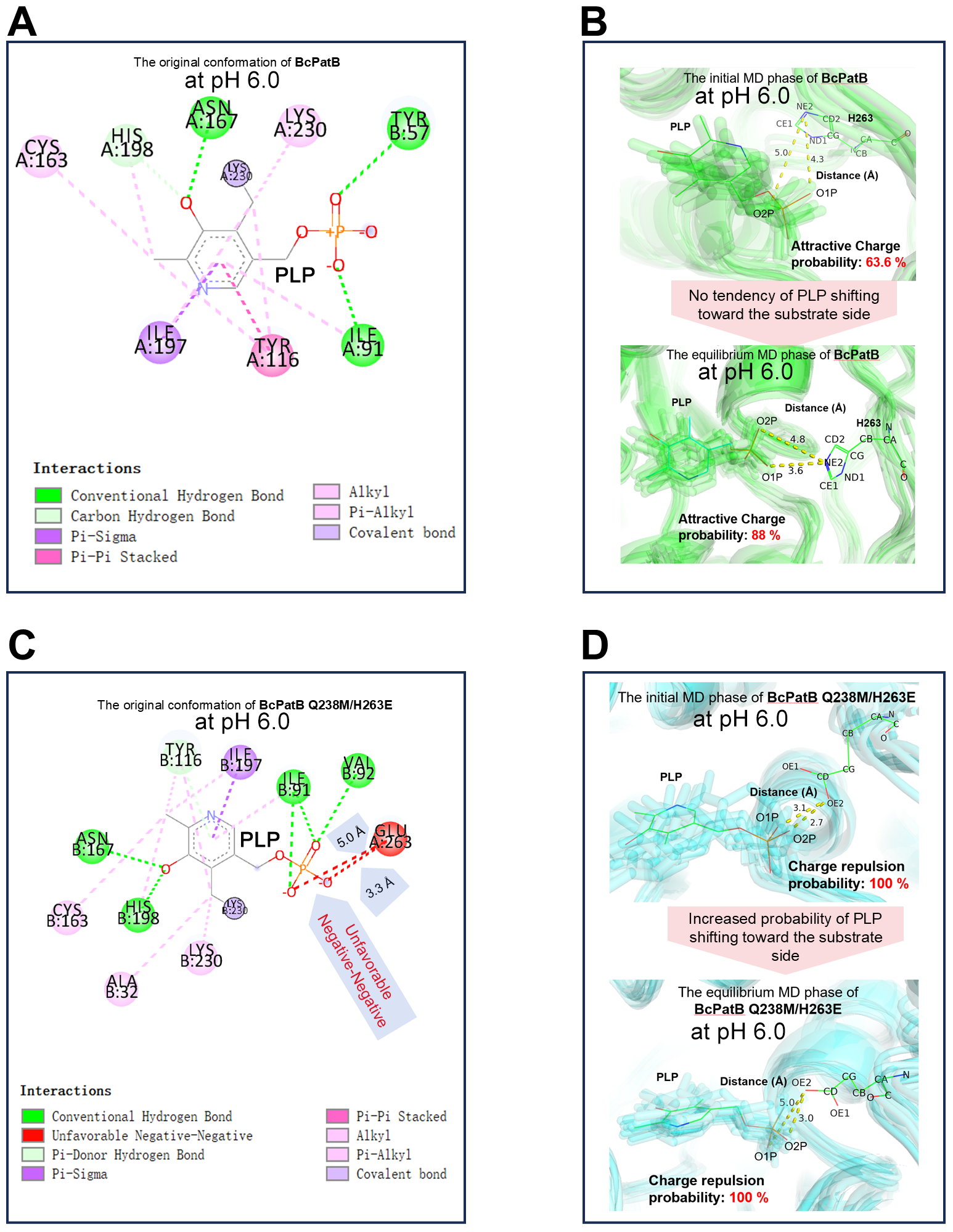
**

**Figure S21. Constant-pH molecular simulations for BcPatB and BcPaB H263E/Q238M** **at pH 6.0**. (A) The original conformation (frame 0) of BcPaB. (B) The initial (frames 0-10, upper panel) and equilibrium MD phase (frames 175-200, lower panel) of BcPaB. Upper panel shows the distance observed between PLP and H263 in the initial phase, and a 63.6% probability of forming attractive charge interactions; Lower panel shows an 88% probability of forming attractive charge in the equilibrium phase. (C) The original conformation (frame 0) of mutant BcPaB H263E/Q238M. (D) The initial (frames 0-10, upper panel) and equilibrium MD phase (frames 175-200, lower panel) of mutant BcPaB H263E/Q238M. The upper panel shows the distance between PLP and E263 in the initial phase, and a 100% probability of forming charge repulsion interactions; the lower panel shows a constantly charge repulsion with 100% probability in the equilibrium phase. The distances between atoms of PLP and the residue pair are indicated by yellow dashed line.

**
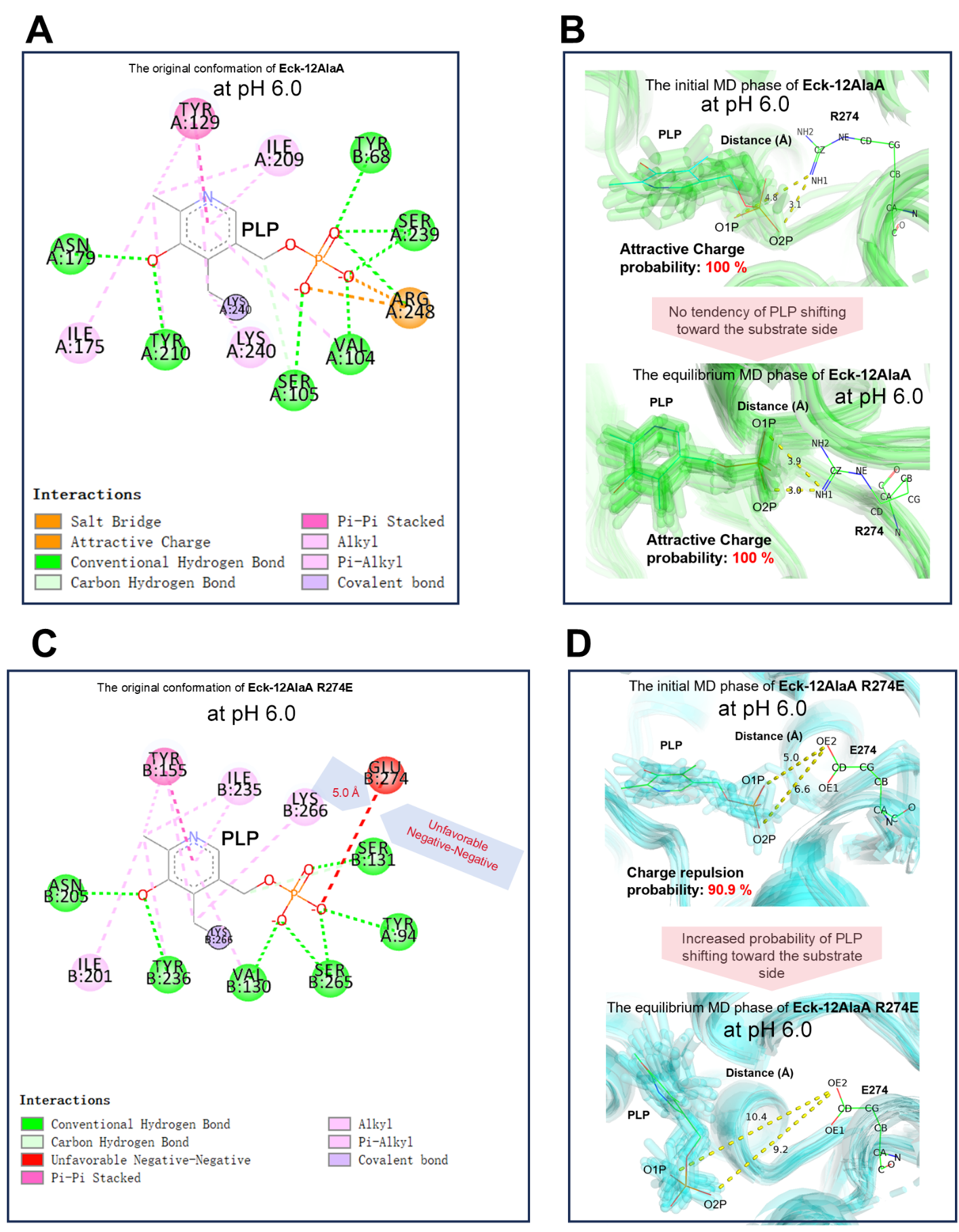
**

**Figure S22. Constant-pH molecular simulations for** **Eck-12AlaA and Eck-12AlaA R274E at pH 6.0**. (A) The original conformation (frame 0) of Eck-12AlaA. (B) The initial (frames 0-10) and equilibrium MD phase (frames 175-200) of Eck-12AlaA. Upper panel shows the distance between PLP and R274 in the initial phase, and a probability (100%) of forming attractive electrostatic interaction; Lower panel shows the distance between PLP and R274 in the equilibrium phase, and a 100% probability of forming attractive electrostatic interaction. (C) The original conformation (frame 0) of mutant Eck-12AlaA R274E. (D) The initial (frames 0-10) and equilibrium MD phase (frames 175-200) of mutant Eck-12AlaA R274E. The upper panel shows the distance between PLP and E274 in the initial phase, with a charge repulsion probability of 90.9%; the lower panel shows a remarkably enlarged distance between PLP and E274 in the equilibrium phase. The distances between atoms of PLP and the residue pair are indicated by yellow dashed line.

**Supplementary Tables 1-8**

**Table S1.** **Minimum substrate concentration for the inhibition of *E. coli* MG1655 in the PatB’s reaction mixture at different pH levels.**

|  | **pH 6.0** | | | **pH 8.0** | | |
| --- | --- | --- | --- | --- | --- | --- |
|  | BcPatB | MePatB | BcPatB  H263E/  Q238M | BcPatB | MePatB | BcPatB  H263E/  Q238M |
| Substrate | l-(±)-alliin | l-(±)-alliin | l-(±)-alliin | l-(±)-alliin | l-(±)-alliin | l-(±)-alliin |
| concentration (μg/mL) | 166.7 ± 72.2 | 0.23 | 3.1 ± 1.1 | 9.1 ± 5.9 | 0.77 ± 0.3 | 12.2 ± 4.2 |

**Note:** Inhibition was assessed using a two-fold serial dilution in 96-well plates at 37 ^o^C. Each well (100 µL medium) contained 20 µg of purified PatB enzyme. The substrate concentration varied across the wells, with substrate being serially diluted two-fold. After incubation, the substrate concentration in the last clear, bacteria-free well was recorded as the minimum substrate concentration. All experiments were performed in triplicate.

**Table S2. Kinetic parameters of LdPatB and MePatB toward the substrate.**

|  | **LdPatB** | | **MePatB** | |
| --- | --- | --- | --- | --- |
|  | l-(+)-alliin | l-(±)-alliin | l-(+)-alliin | l-(±)-alliin |
| *V*_max_ (μmol pyruvate  min^−1^ mg protein^−1^) | 204.8 ± 8.4 | 187.4 ± 7.6 | 267.2 ± 8.1 | 231.4 ± 7.4 |
| *k*_cat_ (s^−1^) | 150.3 ± 6.0 | 137.5 ± 4.9 | 207.1 ± 5.8 | 179.3 ± 6.7 |
| *K*_m_ (mM) | 12.3 ± 1.4 | 11.4 ± 1.3 | 13.6 ± 1.1 | 13.7 ± 1.2 |
| *k*_cat/_*K*_m_ (10^3^ M^−1^ s^−1^) | 12.2 ± 1.5 | 12.1 ± 1.5 | 15.3 ± 1.4 | 13.1 ± 1.3 |

**Note:** Reactions were performed with 0.1 mM PLP supplementation. Data for MePatB was measured at pH 6.0, while LdPatB was measured at pH 8.0, both at 35 ^o^C. All experiments were performed in triplicate.

**Table S3. Data collection and refinement statistics for the LdPatB structure (PDB ID: 8Y54).**

| **Data collection** | | **Data** |
| --- | --- | --- |
|  | Wavelength (Å) | 0.97904 |
|  | Space group | C 1 2 1 |
|  | Cell dimensions  a, b, c (Å)  α, β, γ (°) | 112.47 Å 91.06 Å 80.87 Å  90.0◦ 125.27◦ 90.0◦ |
|  | Resolution range^a^ (Å) | 38.95–1.78 (1.83‒1.78) |
|  | Total Reflections | 413153 |
|  | Unique Reflections | 63450 (4699) |
|  | Multiplicity | 6.5 (6.7) |
|  | Average (I/σ) | 13.2 (1.5) |
|  | Completeness (%) | 99.6 (99.9) |
|  | R_merge_^b^ | 0.079 (1.17) |
| **Refinement statistics** | |  |
|  | Reflections used in refinement | 63258 |
|  | R_work_^c^ / R_free_^d^ | 0.206/0.234 |
|  | Number of atoms | 6490 |
|  | Protein | 6148 |
|  | PLP | 0 |
|  | Water molecules | 342 |
|  | Average *B* factor [A^2^ ] | 30.0 |
|  | rmsd from ideal |  |
|  | Bond length (Å) | 0.49 |
|  | Bond angles (°) | 0.68 |
|  | Ramachandran plot (%) |  |
|  | Favored/Allowed/Outliers | 97.0/3.0/0.0 |
|  | Rotamer outliers (%) | 1.0 |
|  | Clashscore | 2.0 |

**Note**: ^a^ statistics for the highest-resolution shell are shown between brackets

^b^ Rmerge = Σhkl Σi | Ihkl, i - <Ihkl > | / Σhkl <Ihkl >

^c^ Rwork = (Σ | |Fo| − |Fc| |) / (Σ | |Fo|)

^d^ 5% of data were used for R free calculation

**Table S4. Data collection and refinement statistics for the LdPatB/PLP structure (PDB ID: 9JVA).**

| **Data collection** | | **Data** |
| --- | --- | --- |
|  | Wavelength (Å) | 0.97904 |
|  | Space group | C 1 2 1 |
|  | Cell dimensions  a, b, c (Å)  α, β, γ (°) | 115.66Å 94.22Å 84.76Å  90.0*◦* 126.97*◦* 90.0*◦* |
|  | Resolution range^a^ (Å) | 33.86–1.56 (1.64‒1.56) |
|  | Total Reflections | 360530 |
|  | Unique Reflections | 65530 (1348) |
|  | Multiplicity | 5.5 (5.5) |
|  | Average (I/σ) | 10.0 (1.5) |
|  | Completeness (%) | 99.6 (99.9) |
|  | R_merge_^b^ | 0.1 (0.534) |
| **Refinement statistics** | |  |
|  | Reflections used in refinement | 64678 |
|  | R_work_^c^/R_free_^d^ | 0.166/0.194 |
|  | Number of atoms | 6855 |
|  | Protein | 6156 |
|  | PLP | 32 |
|  | Water molecules | 667 |
|  | Average *B* factor [A^2^ ] | 20.0 |
|  | RMSD from ideal |  |
|  | Bond length (Å) | 0.43 |
|  | Bond angles (°) | 0.60 |
|  | Ramachandran plot (%) |  |
|  | Favored/Allowed/Outliers | 98.0/2.0/0.0 |
|  | Rotamer outliers (%) | 1.0 |
|  | Clashscore | 1.0 |

**Note**: ^a^ statistics for the highest-resolution shell are shown between brackets

^b^ Rmerge = Σhkl Σi | Ihkl, i - <Ihkl > | / Σhkl <Ihkl >

^c^ Rwork = (Σ| |Fo|−|Fc| |) / (Σ|Fo|)

^d^ 5% of data were used for R free calculation

**Table S5. Data collection and refinement statistics for the BcPatB/PLP structure (PDB ID:9K71).**

| **Data collection** | | **Data** |
| --- | --- | --- |
| Wavelength (Å) | | 0.97904 |
|  | Space group | P 21 21 21 |
|  | Cell dimensions  a, b, c (Å)  α, β, γ (°) | 53.42Å 84.65Å 163.38Å  90.0◦ 90.0◦ 90.0◦ |
|  | Resolution range^a^ (Å) | 42.33–2.28 (2.40–2.28) |
|  | Total Reflections | 383744 |
|  | Unique Reflections | 34049 (4764) |
|  | Multiplicity | 11.3 (9.7) |
|  | Average (I/σ) | 10.7 (2.6) |
|  | Completeness (%) | 98.3 (96.1) |
|  | R_merge_^b^ | 0.160 (0.904) |
| **Refinement statistics** | |  |
|  | Reflections used in refinement | 33844 |
|  | R_work_^c^ / R_free_^d^ | 0.229/0.310 |
|  | Number of atoms | 6343 |
|  | Protein (with 2 LLP) | 6237 |
|  | Water molecules | 106 |
|  | Average *B* factor [A^2^ ] | 39.0 |
|  | RMSD from ideal |  |
|  | Bond length (Å) | 0.52 |
|  | Bond angles (°) | 0.69 |
|  | Ramachandran plot (%) |  |
|  | Favored/Allowed/Outliers | 96.0/4.0/0.0 |
|  | Rotamer outliers (%) | 3.0 |
|  | **Clashscore** | **13** |

**Note**: ^a^ statistics for the highest-resolution shell are shown between brackets;

^b^ Rmerge = Σhkl Σi | Ihkl, i - <Ihkl > | / Σhkl <Ihkl >

^c^ Rwork = (Σ | |Fo| − |Fc| |) / (Σ | |Fo|)

^d^ 5% of data were used for R free calculation

**Table S6. Design principles for mutants at the residue pair of various enzymes.**

| **Mutants** | **Designing principles** |
| --- | --- |
| BcPatB H263E | Generate a subtle repulsion above the negatively charged phosphate of PLP |
| BcPatB H263D | Generate a subtle repulsion above the negatively charged phosphate of PLP |
| BcPatB Q238M | Eliminate the potential interaction below the phosphate tail of PLP/mutated into M as acidic MePatB adopted |
| BcPatB H263E/Q238M | Generate a subtle repulsion above the phosphate of PLP and eliminate the potential interaction of glutamine with the phosphate below |
| BcPatB Q238E | Generate a subtle repulsion below the phosphate of PLP |
| BcPatB Q238E/H263E | Generate subtle repulsions both above and below the phosphate of PLP |
| BcPatB Q238R | Generate a potential attraction below the phosphate of PLP |
| LdPatB E267H | Generate a subtle potential attraction above the phosphate of PLP |
| LdPatB E267F | Eliminate the potential repulsion above the phosphate of PLP |
| LdPatB H241R | Generate an increased potential attraction below the phosphate of PLP |
| LdPatB H241M | Eliminate the potential attraction below the phosphate tail of PLP/mutated into M as acidic MePatB adopted |
| LdPatB H241M/E267F | Eliminate the potential attraction below the phosphate of PLP and eliminate the potential repulsion above the phosphate of PLP |
| LdPatB H241E | Convert the potential attraction below the phosphate tail of PLP to a potential repulsion |
| MePatB F279E | Generate a potential repulsion above the phosphate of PLP |
| MePatB F279R | Generate a potential attraction above the phosphate of PLP |
| MePatB F279W | Increase the potential interaction above the phosphate of PLP |
| MePatB F279Y | Increase the potential interaction above the phosphate of PLP |
| MePatB M255E | Generate a potential repulsion below the phosphate of PLP |
| MePatB M255E/F279E | Generate potential repulsions both above and below the phosphate of PLP |
| MePatB M255R | Generate a potential attraction below the phosphate of PLP |
| KpMetC G241H | Generate a potential attraction above the phosphate of PLP |
| KpMetC G241E | Generate a potential repulsion above the phosphate of PLP |
| KpMetC G241D | Generate a potential repulsion above the phosphate of PLP |
| KpMetC G241S | Generate a potential interaction above the phosphate of PLP |
| KpMetC G241A | Investigate the importance of this site for structural and functional aspects |
| KpMetC M219R | Generate a potential attraction below the phosphate of PLP |
| KpMetC M219E | Generate a potential repulsion below the phosphate of PLP |
| Eck-12AlaA R274M | Eliminate the potential attraction below the phosphate of PLP mutated into M as acidic MePatB adopted |
| Eck-12AlaA R274E | Generate a potential repulsion below the phosphate of PLP |
| Eck-12AlaA L302R | Generate a potential attraction above the phosphate of PLP |
| Eck-12AlaA L302E | Generate a potential repulsion above the phosphate of PLP |
| Eck-12AlaA L302E/R274E | Generate potential repulsions both above and below the phosphate of PLP |

**Table S7. Buffers used in this study.**

| **Buffers** | **Ingredients** (L^-1^) |
| --- | --- |
| Protein lysis buffer | Tris-HCl buffer, 3.03 g Tris, 8.08 g NaCl, pH 8.0 |
| Molecular sieve buffer | Tris-HCl buffer, 3.03 g Tris, 8.08 g NaCl, pH 8.0 |
| Nickel column A solution | Tris-HCl buffer, 3.03 g Tris, 29.25 g NaCl, pH 8.0 |
| Nickel column B solution | Tris-HCl buffer, 3.03 g Tris, 29.25 g NaCl,  34.03 g imidazole, pH 8.0 |
| Na_2_HPO_4_-CA buffer | 5.84 g Na_2_HPO_4_, 33.37 g citric acid, pH 3.0 |
| Na_2_HPO_4_-CA buffer | 10.94 g Na_2_HPO_4_, 25.81 g citric acid, pH 4.0 |
| Na_2_HPO_4_-CA buffer | 14.61 g Na_2_HPO_4_, 20.36 g citric acid, pH 5.0 |
| Na_2_HPO_4_-CA buffer | 17.94 g Na_2_HPO_4_, 15.48 g citric acid, pH 6.0 |
| Na_2_HPO_4_-CA buffer | 23.38 g Na_2_HPO_4_, 7.42 g citric acid, pH 7.0 |
| Na_2_HPO_4_-CA buffer | 27.58 g Na_2_HPO_4_, 1.16 g citric acid, pH 8.0 |

**Table S8. Oligonucleotide primers engineered for site-directed mutagenesis.**

| **Mutant** | **Primer F** | **Primer R** |
| --- | --- | --- |
| **BcPatB** |  |  |
| H263E | ACAGGGCTTCGAAGGGTTAAATATCTTTGCTT | TTAACCCTTCGAAGCCCTGTCTATACTGGA |
| H263D | ACAGGGCTTCGATGGGTTAAATATCTTTGCTT | TTAACCCATCGAAGCCCTGTCTATACTGGA |
| Q238M | TGGATTAATGGCGTCGATTATTATCATTC | TAATCGACGCCATTAATCCAGCGATATTAAAT |
| A32K | TGCTTGGATTAAAGATATGGATTTTGAAGTACC | CCATATCTTTAATCCAAGCATGGATAAGTT |
| A32D | TGCTTGGATTGATGATATGGATTTTGAAGTACC | CCATATCATCAATCCAAGCATGGATAAGTT |
| N167K | CAGTCCTCACAAACCGATAGGGCGTGTTTGGAC | CTATCGGTTTGTGAGGACTGCAAAGAAGCATGAG |
| N167E | CAGTCCTCACGAACCGATAGGGCGTGTTTGGAC | CTATCGGTTCGTGAGGACTGCAAAGAAGCATGAG |
| H263K | ACAGGGCTTCAAAGGGTTAAATATCTTTGCTT | TTAACCCTTTGAAGCCCTGTCTATACTGGA |
| I31R | TGCTTGGCGTGCTGATATGGATTTTGAAGT | CCATATCAGCACGCCAAGCATGGATAAGTTCT |
| D33N | GGATTGCTAATATGGATTTTGAAGTACC | AAAATCCATATTAGCAATCCAAGCATGGATAA |
| Y57H | ATTTTTGGCCATACACTTCCTCCCGAAAATAT | GGAAGTGTATGGCCAAAAATCGGATGTT |
| Y116H | CACCTATTCATCCTCCATTTTTCGAAATGGT | AAATGGAGGATGAATAGGTGGTTGTACGAGAA |
| Y352H | GTGAGAAGCATGGATTAGGCGGCGAAGAACA | GCCTAATCCATGCTTCTCACCAGGTTCAACGA |
| I31E | TGCTTGGGAAGCTGATATGGATTTTGAAGT | CCATATCAGCTTCCCAAGCATGGATAAGTTCT |
| Y57E | ATTTTTGGCGAAACACTTCCTCCCGAAAATAT | GGAAGTGTTTCGCCAAAAATCGGATGTT |
| Y352E | GTGAGAAGGAAGGATTAGGCGGCGAAGAACA | GCCTAATCCTTCCTTCTCACCAGGTTCAACGA |
| Q238E | TGGATTAGAAGCGTCGATTATTATCATTC | TAATCGACGCTTCTAATCCAGCGATATTAAAT |
| Q238R | TGGATTACGCGCGTCGATTATTATCATTC | TAATCGACGCGCGTAATCCAGCGATATTAAAT |
| **LdPatB** |  |  |
| E267H | TATCGGGCATCCAAACTTGTTGGCTATCCC | ACAAGTTTGGATGCCCGATACCGGCCAGGAAAA |
| E267F | TATCGGGTTTCCAAACTTGTTGGCTATCC | ACAAGTTTGGAAACCCGATACCGGCCAGGAAA |
| H241R | GGCGTTGCGCGCGGCTTGCGCGATTATC | CGCAAGCCGCGCGCAACGCCGCCAAGTTGAAG |
| H241M | GGCGTTGATGGCGGCTTGCGCGATTATC | CGCAAGCCGCCATCAACGCCGCCAAGTTGAAG |
| H241E | GGCGTTGGAAGCGGCTTGCGCGATTATC | CGCAAGCCGCTTCCAACGCCGCCAAGTTGAAG |
| **MePatB** |  |  |
| F279E | TGTGGGAGAAATCAATCCCTTGTCCCTCGA | AGGGATTGATTTCTCCCACAGTCTTGTCGCGG |
| F279R | TGTGGGACGCATCAATCCCTTGTCCCTCGA | AGGGATTGATGCGTCCCACAGTCTTGTCGCGG |
| F279W | TGTGGGATGGATCAATCCCTTGTCCCTCGA | AGGGATTGATCCATCCCACAGTCTTGTCGCGG |
| F279Y | TGTGGGATATATCAATCCCTTGTCCCTCGA | AGGGATTGATATATCCCACAGTCTTGTCGCGG |
| M255E | CGGCCTCGAATTCTCGGACATCATCATC | TGTCCGAGAATTCGAGGCCGGCCATGTTGAA |
| M255R | CGGCCTCCGCTTCTCGGACATCATCATC | TGTCCGAGAAGCGGAGGCCGGCCATGTTGAA |
| **KpMetC** |  |  |
| G241H | CCTGATGCATCAAATGCTGGACGCCGATA | CCAGCATTTGATGCATCAGGTAGGCATTTTCAC |
| G241E | CCTGATGGAACAAATGCTGGACGCCGATA | CCAGCATTTGTTCCATCAGGTAGGCATTTTCAC |
| G241D | CCTGATGGATCAAATGCTGGACGCCGATA | CCAGCATTTGATCCATCAGGTAGGCATTTTCAC |
| G241S | CCTGATGAGCCAAATGCTGGACGCCGATA | CCAGCATTTGGCTCATCAGGTAGGCATTTTCAC |
| G241A | CCTGATGGCGCAAATGCTGGACGCCGATA | CCAGCATTTGCGCCATCAGGTAGGCATTTTCAC |
| M219R | CGACGCCCGCGTGGGCACCGCGGTGG | CGGTGCCCACGCGGGCGTCGGAATGGCCG |
| M219E | CGACGCCGAAGTGGGCACCGCGGTGG | CGGTGCCCACTTCGGCGTCGGAATGGCCG |
| **Eck-12AlaA** |  |  |
| R274M | TGCTGGTTTCATGCAGGGCTGGATGGTTCT | AGCCCTGCATGAAACCAGCAACACGG |
| R274E | TGCTGGTTTCGAACAGGGCTGGATGGTTCT | AGCCCTGTTCGAAACCAGCAACACGG |
| L302R | TATGCGTCGCTGCGCGAACGTACCGGC | CGTTCGCGCAGCGACGCATAGAAGCCAGCAT |
| L302E | TATGCGTGAATGCGCGAACGTACCGGC | CGTTCGCGCATTCACGCATAGAAGCCAGCAT |

**Protein sequences used in this study**

**BcPatB**

MQLFHKAINRRGTHSIKWDTYKNEELIHAWIADMDFEVPKPIQTALKQRIEHPIFGYTLPPENIGDIICNWTKQQYNWDIQKEWIVFSAGIVPALSTSIQAFTKENESVLVQPPIYPPFFEMVTTNNRQLCVSPLQKQNDTYVIDFKHLEKQFQQGIKLLLLCSPHNPIGRVWTKEELVQLGSLCTKYDVIVVADEIHSDIIYADHTHTPFASLSEELAERTITCMAPSKTFNIAGLQASIIIIPNEKLRHAFTAIQYRQGFHGLNIFAYTAMQSAYTECNDWLNEIRLYIEDNAQFACEYIKTHIPALSVTKPEGSFLLWIDCSRLKLSQNERTALLEEKGKIIVEPGEKYGLGGEEHIRINIGCPRSVLEEILNRLRHTFS

**LdPatB**

MAEKQYDFTHVPKRQGNSIKWGGLKEKELPMWIAEMDFRIAPEIMTSMEEKLKVAAFGYESVPAEYYKAVADWEEIEHRARPKEDWCVFASGVVPAISAMVRQFTSPGDQILVQEPVYNMFYSVIEGNGRRVISSDLIYENSKYSVNWADLEEKLATPSVRMMVFCNPHNPIGYAWSEEEVKRIAELCAKHQVLLISDEIHGDLVLTDEDITPAFTVDWDAKNWVVSLISPSKTFNLAALHAACAIIPNPDLRARAEESFFLAGIGEPNLLAIPAAIAAYEEGHNWLRELKQVLRDNFAYAREFLAKEVPEVKVLDSNASYLAWVDISALGMNAEDFCKYLREKTGLIISAGNGYRGNGHEFVRINLACPKELVIDGMQRLKQGVLNLNN

**MePatB**

MTYDFDEIIDRRHTNALNTDGFRGYIFHAGPEKKFPFADDEFVRMWVADMEFATPPEICQAMKDRIDRRIFGYTLLCDDSYYDVFAGWCQKMYGWTFPKEELVFSPGIIPALYELVEDLVKDTEKILMVSPAYGFFQHAAEYADRTYVCSPLMYDAGKFSIDFDDFEAKAADPHMKLVIWCNPHNPTGRIWTEEELRRVAAIVEKYNLWIISDEIHCDLLRCGKTHTPMGKIMADYPKLVTCMSASKTFNMAGLMFSDIIIRDAELRRHFAARDKTVGFINPLSLEAHKAAYLSCSDWLDQLRAYLDGNFQFVKDYVAEHLPKISFEIPDATYLAWMDMSPYLGDVENIPDFFANEAGVLLEGGDSLFVGNAKGFIRLNLAMPRSIIAEGLKRMDEAIQKHLATK

**KpMetC**

MADKHLDTALVNAGRSKKYTQGSVNSVIQRASSLVFDTVEAKKHATRNRANGELFYGRRGTLTHFSLQEAMCELEGGAGCALFPCGAAAVANTILAFVEQGDHVLMTNTAYEPSQDFCTKILAKLGVTTSWFDPLIGADIARLVRPETRVVFLESPGSITMEVHDVPAIVAAVRQVAPEAIIMIDNTWAAGILFKALDFGIDISIQAGTKYLIGHSDAMVGTAVANARCWPQLRENAYLMGQMLDADTAYMTSRGLRTLGVRLRQHHESSLRIAEWLAQHPQVARVNHPALPGSKGHEFWKRDFTGSSGLFSFVLSKRLNDAELAEYLDNFSLFSMAYSWGGFESLILANQPEQIAHIRPDAEVDFSGTLDRLHIGLENVDDLQADLAAGFARIV

**Eck-12AlaA**

MKHHHHHHPMSDYDIPTTENLYFEGAMSPIEKSSKLENVCYDIRGPVLKEAKRLEEEGNKVLKLNIGNPAPFGFDAPDEILVDVIRNLPTAQGYCDSKGLYSARKAIMQHYQARGMRDVTVEDIYIGNGVSELIVQAMQALLNSGDEMLVPAPDYPLWTAAVSLSSGKAVHYLCDESSDWFPDLDDIRAKITPRTRGIVIINPNNPTGAVYSKELLMEIVEIARQHNLIIFADEIYDKILYDDAEHHSIAPLAPDLLTITFNGLSKTYRVAGFRQGWMVLNGPKKHAKGYIEGLEMLASMRLCANVPAQHAIQTALGGYQSISEFITPGGRLYEQRNRAWELINDIPGVSCVKPRGALYMFPKIDAKRFNIHDDQKMVLDFLLQEKVLLVQGTAFNWPWPDHFRIVTLPRVDDIELSLSKFARFLSGYHQL
